# Quantifying *Plasmodium vivax* radical cure efficacy: a modelling study integrating clinical trial data and transmission dynamics

**DOI:** 10.1016/S1473-3099(24)00689-3

**Published:** 2025-06

**Authors:** Constanze Ciavarella, Chris Drakeley, Ric N Price, Ivo Mueller, Michael White

**Affiliations:** a Institut Pasteur, Université Paris Cité, G5 Épidémiologie et Analyse des Maladies Infectieuses, Paris, France; b London School of Hygiene & Tropical Medicine, London, UK; c Global and Tropical Health Division, Menzies School of Health Research and Charles Darwin University, Darwin, NT, Australia; d Centre for Tropical Medicine and Global Health, Nuffield Department of Medicine, University of Oxford, Oxford, UK; e Mahidol-Oxford Tropical Medicine Research Unit, Faculty of Tropical Medicine, Mahidol University, Bangkok, Thailand; f Walter and Eliza Hall Institute, Parkville, VIC, Australia

## Abstract

**Background:**

*Plasmodium vivax* forms dormant liver stages (hypnozoites) that can reactivate weeks to months after primary infection. Radical cure requires a combination of antimalarial drugs to kill both the blood-stage and liver-stage parasites. Hypnozoiticidal efficacy of the liver-stage drugs primaquine and tafenoquine cannot be estimated directly because hypnozoites are undetectable. We aimed to estimate hypnozoiticidal efficacy from clinical trial data, and quantify the community-level impact of implementing case management with radical cure.

**Methods:**

We calibrated a novel *P vivax* Recurrence Model to publicly available data from prospective clinical trials to estimate the hypnozoiticidal efficacy of different supervised primaquine (3·5 mg/kg or 7 mg/kg over 7 or 14 days) and tafenoquine (5 mg/kg or 7·5 mg/kg single dose) regimens in patients with normal glucose-6-phosphate dehydrogenase (G6PD) activity. We used an existing *P vivax* Individual-Based Model to quantify the 5-year impact of case management with unsupervised primaquine or tafenoquine regimens across various transmission settings.

**Findings:**

We estimated median hypnozoiticidal efficacies of 99·1% (95% credible interval 96·0–100) for primaquine 7 mg/kg over 14 days; 96·3% (90·8–99·7) for primaquine 7 mg/kg over 7 days; 72·3% (68·1–76·3) for primaquine 3·5 mg/kg over 7 or 14 days; 62·4% (49·1–76·3) for tafenoquine 5 mg/kg single dose; and 87·5% (62·1–99·3) for tafenoquine 7·5 mg/kg single dose. 5 years of community-level tafenoquine case management was estimated to reduce *P vivax* transmission by 74–79% where pre-intervention prevalence as measured by PCR was low (<2%) and by 17–20% where prevalence as measured by PCR was high (around 35%). Similar 5-year reductions were estimated with primaquine case management only when adherence to the primaquine regimen was above 50%.

**Interpretation:**

Substantial reductions in prevalence as measured by PCR were predicted with primaquine and tafenoquine regimens if these could be implemented with high coverage and adherence. The benefits of preventing *P vivax* relapses need to be balanced against the risks of inducing severe haemolysis in patients with G6PD deficiency.

**Funding:**

Bill & Melinda Gates Foundation and Horizon Europe.

## Introduction

*Plasmodium vivax* is a major cause of malaria in tropical regions. In 2022, its burden was estimated at 6·9 million clinical cases globally.^1^ *P vivax* malaria is characterised by the ability of the parasite to form dormant liver stages (known as hypnozoites), which can reactivate weeks to months after the initial infection to cause recurrent episodes of parasitaemia (known as relapses). Relapses are estimated to be responsible for 66–95% of cases of *P vivax* malaria in endemic areas.^2^, ^3^ Therefore, the eradication of latent hypnozoites is a high priority for *P vivax* malaria control.^4^

Blood-stage (schizontocidal) antimalarial drugs, such as chloroquine or artemisinin-based combination therapy, kill the *P vivax* blood-stage parasites that cause febrile illness, but have no activity against hypnozoites that cause relapses. The only drugs capable of killing hypnozoites are the 8-aminoquinolines primaquine and tafenoquine. Both 8-aminoquinoline compounds can cause severe haemolysis in individuals with glucose-6-phosphate dehydrogenase (G6PD) deficiency, an inherited enzymopathy, present in up to 30% of the population in malaria-endemic countries.^5^

Point-of-care tests with high sensitivity and high specificity are now available to evaluate G6PD enzyme activity in patients before prescribing 8-aminoquinolines.^6^ Although these tests can reduce the risk of haemolysis, their application in remote and poorly resourced areas is logistically challenging.^6^ The benefits of preventing *P vivax* relapses need to be balanced against the risks of inducing severe haemolysis in patients with G6PD deficiency.^7^, ^8^

Recurrent parasitaemia can arise from either recrudescence (due to schizontocidal treatment failure), relapse (due to hypnozoite reactivation), or re-infection (from a new mosquito inoculation). Estimates of 8-aminoquinoline efficacy based on the number of recurrences observed in the intervention group versus the control group of a clinical trial are biased downwards by recrudescence and re-infection. To minimise these biases, clinical trials might exclude patients with suspected schizontocidal treatment failure,^9^ and prevent re-infection through extensive vector control campaigns or by moving participants to non-endemic areas during the follow-up period.^10^ The cause of recurrent *P vivax* might also be resolved probabilistically by genotyping parasites in blood samples of primary and recurrent infections.^3^

**Research in context**

**Evidence before this study**

We searched PubMed on Jan 31, 2024, for studies using mathematical modelling to estimate the hypnozoiticidal efficacy of primaquine and tafenoquine regimens using the search terms (“8-aminoquinoline” OR “primaquine” OR “tafenoquine”) AND “vivax” AND (“modelling” OR “modeling”), with no language or date restrictions. Of the 32 articles returned, only two estimated the hypnozoiticidal efficacy of 7 mg/kg total of supervised primaquine given over 7 or 14 days. One study estimated a hypnozoiticidal efficacy of 97·1% (95% credible interval 96·2–97·7) for primaquine (7 mg/kg total over 7 or 14 days) given in combination with chloroquine (25 mg/kg total over 3 days) or dihydroartemisinin-piperaquine (7 mg/kg dihydroartemisinin and 55 mg/kg piperaquine total over 3 days) to patients older than 6 months on the Thailand–Myanmar border. Another study estimated a hypnozoiticidal efficacy of 60% (95% CI 52–68) for primaquine (7 mg/kg total over 14 days) given in combination with artesunate (28 mg/kg total over 7 days) to Papua New Guinean children between 1 and 5 years of age. The remaining 30 articles were not relevant.

**Added value of this study**

We estimated the hypnozoiticidal efficacy of a range of primaquine and tafenoquine regimens, varying dose and duration of administration. Case management with both low-dose and high-dose primaquine and tafenoquine regimens is predicted to reduce transmission substantially. Where there are safety concerns for high-dose regimens, similar reductions in prevalence as measured by PCR were predicted with low-dose regimens if these could be implemented with high levels of coverage and adherence. Once transmission has been reduced and experience with administering primaquine or tafenoquine acquired, a switch to high-dose regimens could be adopted to push towards *Plasmodium vivax* elimination.

**Implications of all the available evidence**

Our findings will enable clinicians and national malaria control programmes to communicate probable treatment outcomes to individual patients and make informed decisions on what primaquine or tafenoquine regimen to implement. Patient-level and community-level impacts described in this study should be carefully evaluated along with complementary data on drug safety and tolerability, drug adherence and acceptability, glucose-6-phosphate dehydrogenase (G6PD) testing, and cost-effectiveness of interventions. Where complementary safety data are insufficient and G6PD testing cannot be assured, a conservative approach should be taken favouring safer, lower-dose regimens. We highlight the opportunity to maximise radical cure impact by improving primaquine adherence and widening 8-aminoquinoline eligibility criteria.

A meta-analysis of data from clinical trials of radical cure with primaquine estimated that there were 1·6 times more recurrent infections after administration of low-dose primaquine (3·5 mg/kg) compared with high-dose primaquine (7 mg/kg).^11^ Similar findings were observed in a meta-analysis of data from clinical trials of radical cure with tafenoquine, in which low-dose tafenoquine (5 mg/kg) resulted in 2·9 times more recurrent infections compared with high-dose tafenoquine (7·5 mg/kg).^12^ Decisions on antimalarial policy need to be guided by the evidence of increased antirelapse efficacy with higher doses of drug and the associated risks of haemolysis.

We aimed to apply mathematical modelling techniques to evaluate the effects of implementing radical cure (combination of blood-stage and liver-stage antimalarial drugs) on patient-level and community-level *P vivax* transmission.

## Methods

### Study design

Our analysis was structured in two parts. We first developed a novel mathematical approach to estimate the hypnozoiticidal efficacy of primaquine and tafenoquine regimens (varying dosage and duration of administration) from prospective clinical trial data on observed *P vivax* recurrences. We then used estimates of hypnozoiticidal efficacy to simulate the patient-level and community-level impact of radical cure with different primaquine and tafenoquine regimens (co-administered with chloroquine for schizontocidal activity) under operational conditions. We quantified the patient-level impact by accounting for 8-aminoquinoline eligibility (ie, G6PD activity, pregnancy, breastfeeding, and younger age) and for primaquine adherence. We estimated the community-level impact across a range of transmission settings (varying transmission intensity, seasonality, *P vivax* relapse rates, and health-care-seeking behaviour) using a previously published model of *P vivax* transmission.^13^, ^14^

### Estimating hypnozoiticidal efficacy

We accessed publicly available data from prospective clinical trials of various radical cure regimens (details are shown in appendix pp 3–4). The IMPROV clinical trial^9^ recruited 2359 patients with more than 30% G6PD activity who were randomly assigned to one of three groups: 7 mg/kg of primaquine over 7 days, 7 mg/kg of primaquine over 14 days, or placebo. A meta-analysis by Commons and colleagues^11^ included 6850 patients with more than 30% G6PD activity who were divided into one of three groups: 3·5 mg/kg of primaquine, 7 mg/kg of primaquine, or control (placebo or no 8-aminoquinoline). Another meta-analysis of placebo-controlled clinical trials by Watson and colleagues^12^ included 861 patients with more than 70% G6PD activity who were divided into four groups: 3·5 mg/kg of primaquine over 14 days, 5 mg/kg of tafenoquine, 7·5 mg/kg of tafenoquine, or placebo. Cumulative incidence risks were estimated by Kaplan–Meier analysis stratified by 8-aminoquinoline regimen and, for the IMPROV clinical trial, by location.

The *P vivax* Recurrence Model (PvRM) is a deterministic compartmental model of recurrent *P vivax* blood-stage infections in symptomatic patients in trials who were treated with antimalarial drugs. Before treatment, symptomatic patients are assumed to have ongoing blood-stage and liver-stage infections. After drug treatment, residual levels of antimalarial drugs protect patients against subsequent recurrent infections for a duration, *d*, which depends on the drug regimen (appendix p 6). This effect is called post-treatment prophylaxis. A full course of an 8-aminoquinoline regimen is assumed to have hypnozoiticidal efficacy, ε, such that all hypnozoites are eliminated in a fraction, ε, of patients, while the remaining fraction, 1 – ε, of patients carry their original hypnozoites (all-or-nothing mechanism of hypnozoite elimination). The fraction ε of patients without hypnozoites after treatment completion get re-infected at a rate λ after the end of post-treatment prophylaxis. The fraction 1 – ε of patients who carry hypnozoites after treatment clear hypnozoites at a rate γ, and, following the end of post-treatment prophylaxis, relapse at a rate *f*, and get re-infected at a rate λ. The PvRM accounts for primaquine failure in patients who are low cytochrome P450 2D6 (CYP2D6) metabolisers (tafenoquine is not affected by this). For details on the PvRM see appendix pp 4–7.

Parameters λ, *f*, and ε are unknown quantities that are determined by fitting the PvRM to clinical trial data using Bayesian Markov Chain Monte Carlo methods. Distinct relapse (*f*) and infection (λ) rates were fitted for each location, and distinct hypnozoiticidal efficacies (ε) were fitted for each 8-aminoquinoline regimen (appendix pp 6–12). We conducted sensitivity analyses on the values of fixed parameters (duration of post-treatment prophylaxis, prevalence of low CYP2D6 metabolisers, and rate of hypnozoite clearance), the mechanism of hypnozoite elimination, and subsets of clinical trial data (appendix p 6). For all scenarios, we calculated the 95% credible interval (CrI) of each fitted parameter as the 2·5th percentile and the 97·5th percentile of its posterior probability distribution.

### Simulating the patient-level effect of radical cure

Primaquine is most commonly prescribed at 3·5 mg/kg or 7 mg/kg, with doses spread evenly over 7 or 14 days (table 1). We considered regimens of 5 mg/kg or 7·5 mg/kg of tafenoquine to be taken all at once. We also analysed a hypothetical perfect 8-aminoquinoline, a so-called magic bullet, which we assumed requires a single dose, does not have any contraindications, and has perfect hypnozoiticidal efficacy. Under trial conditions, we assumed drug adherence to be perfect. Conversely, we assumed that in routine clinical practice patients take the first 8-aminoquinoline dose under supervision and thus adherence to the single-dose drug tafenoquine is perfect, whereas we assumed 67% adherence to 7-day primaquine and 57% adherence to 14-day primaquine (these values were varied in the sensitivity analyses; appendix p 20).

**Table 1:** Overview of investigated 8-aminoquinoline regimens

	**8-aminoquinoline**	**Number of days**	**Daily dose, mg/kg**	**Total dose, mg/kg**	**Total dose for a 60 kg patient, mg**	**Demographic eligibility criteria**	G6PD eligibility criteria*
No 8-aminoquinoline	None	0	NA	NA	NA	None	None
Primaquine (3·5 mg/kg over 14 days)	Primaquine	14	0·25	3·5	210	Aged >6 months, not pregnant, not breastfeeding	G6PD activity >30%
Primaquine (3·5 mg/kg over 7 days)	Primaquine	7	0·5	3·5	210	Aged >6 months, not pregnant, not breastfeeding	G6PD activity >30%
Primaquine (7 mg/kg over 14 days)	Primaquine	14	0·5	7	420	Aged >6 months, not pregnant, not breastfeeding	G6PD activity >30%
Primaquine (7 mg/kg over 7 days)	Primaquine	7	1	7	420	Aged >6 months, not pregnant, not breastfeeding	G6PD activity >70%
Tafenoquine (5 mg/kg single dose)	Tafenoquine	1	5	5	300	Aged >2 years, not pregnant, not breastfeeding	G6PD activity >70%
Tafenoquine (7·5 mg/kg single dose)	Tafenoquine	1	7·5	7·5	450	Aged >2 years, not pregnant, not breastfeeding	G6PD activity >70%
Magic bullet (single dose)	Hypothetical	1	NA	NA	NA	None	None

G6PD=glucose-6-phosphate dehydrogenase. NA=not applicable.

* Individuals' G6PD enzyme activity was measured as a percentage of the population median G6PD enzyme activity.

Our analysis assumed that all patients with symptomatic *P vivax* malaria are treated with a course of schizontocidal antimalarial drugs that clears blood-stage parasites but not hypnozoites (ie, 100% schizontocidal adherence and 100% schizontocidal efficacy). Patients are then prescribed an 8-aminoquinoline regimen if they meet the corresponding eligibility criteria on age, pregnancy and breastfeeding status, and G6PD activity (table 1). Following methods described by Nekkab and colleagues,^14^ prevalence of deficient G6PD genotypes was mapped to phenotypic G6PD activity via a Gaussian mixture model. We assumed that patients who are not eligible for tafenoquine or 7-day high-dose primaquine (7 mg/kg) are prescribed 7-day low-dose primaquine (3·5 mg/kg), provided their G6PD activity is more than 30%, they are not pregnant or breastfeeding, and are aged 6 months or older. Patients who are not eligible for an 8-aminoquinoline regimen were assumed to only be given schizontocidal drugs. We assumed that drug dosing is accurate and did not model adverse events due to drug treatment.

We defined the average per-patient hypnozoiticidal effectiveness of an 8-aminoquinoline regimen under trial conditions (TC) as

$$e_{\text{TC}}=\{\begin{array}{ll} ɛ(1-c) & \text{if}\;\text{primaquine}\;\text{is}\;\text{given}\\ ɛ & \text{if}\;\text{tafenoquine}\;\text{or}\;\text{magic}\;\text{bullet}\;\text{is}\;\text{given} \end{array}$$

where ε is the median hypnozoiticidal efficacy in participants with normal CYP2D6 metabolism estimated from trial data, and *c* is the prevalence of participants with low CYP2D6 metabolism.

Under operational conditions, the effectiveness of an 8-aminoquinoline regimen is reduced due to eligibility restrictions (table 1) and imperfect adherence to multiday primaquine regimens. Using the synthetic population from the *P vivax* Individual-Based Model (PvIBM) as an example, we estimated that 92% of patients are eligible for primaquine when given at a daily dose of 0·25 mg/kg or 0·5 mg/kg, 80·6% are eligible for primaquine when given at a daily dose of 1 mg/kg, and 75·1% are eligible for tafenoquine (appendix p 15). We defined the average per-patient hypnozoiticidal effectiveness of an 8-aminoquinoline regimen under operational conditions (OC) as

$$e_{\text{oc}}=\{\begin{array}{ll} ue_{\text{TC}}\nu & \text{if}\;\text{no}\;\text{alternative}\;\text{8-aminoquinoline}\;\text{regimen}\;\text{is}\;\text{offered}\\ ue_{\text{TC}}\nu +(\bar{u}-u){\bar{e}}_{\text{TC}}\bar{\nu } & \text{if}\;\text{offering}\;\text{an}\;\text{alternative}\;\text{8-aminoquinoline}\;\text{regimen} \end{array}$$

where *u* denotes the proportion of patients who are eligible for the 8-aminoquinoline regimen and *v* denotes the proportion of patients who adhere to the 8-aminoquinoline regimen. Here, *e*_TC_, *u*, and *v* refer to the primary 8-aminoquinoline regimen, and *ẽ*_TC_, *ũ*, and *ṽ* refer to the alternative 8-aminoquinoline regimen of primaquine (3·5 mg/kg over 7 days).

### Simulating the community-level effect of radical cure case management

The PvIBM simulates *P vivax* relapses, G6PD activity levels by sex, case management, public health interventions, population demography, heterogeneity in exposure to mosquito bites, antiparasite and antidisease immunity, mosquito seasonality, larval mosquito stages, and vector control.^13^, ^14^ We assumed that G6PD screening is carried out using the SD Biosensor STANDARD G6PD test and accounted for misclassification in the PvIBM.^15^ 8-aminoquinoline eligibility criteria (including prescription of secondary 8-aminoquinoline regimens) and adherence to multiday primaquine regimens were included. For details, see appendix pp 17–20.

We simulated the introduction of radical cure into routine case management under the operational conditions described, and quantified its impact at the community level. We assumed transmission to be at equilibrium at the beginning of the PvIBM simulations. Apart from the roll-out of the radical cure case management strategy, we assumed no other changes in public health interventions to take place for the duration of the simulations. Case importation through human or mosquito mobility was not accounted for. We ran simulations across a range of transmission scenarios by varying several key parameters (transmission intensity, seasonality, *P vivax* relapse rates, prevalence of G6PD deficiency, prevalence of patients with low CYP2D6 metabolism, primaquine adherence, case management coverage, and immune response to relapses; appendix p 20). We ran 100 independent simulations for each scenario and output the prevalence as measured by PCR for each timepoint. The point estimate is the median over the 100 simulation runs, and the 95% CI is the 2·5th percentile and the 97·5th percentile of the 100 simulation runs.

### Role of the funding source

The funders of the study had no role in study design, data collection, data analysis, data interpretation, or writing of the report.

## Results

**Figure 1:**
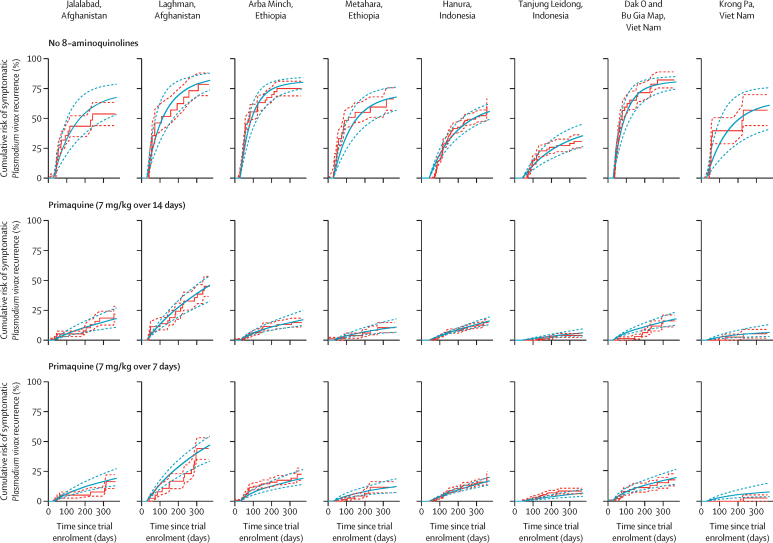
Calibration of the PvRM to patient-level data from the IMPROV clinical trial^9^ using the all-or-nothing mechanism The red solid curves show the Kaplan–Meier estimates for the risk of blood-stage recurrence up to day 365, and the red dashed curves show a standard deviation around the Kaplan–Meier estimates. The solid blue curves show the posterior median model prediction, and the dashed blue curves show the 95% credible intervals. All patients were treated with blood-stage drugs. The PvRM uses hypnozoiticidal efficacy estimates provided in table 2, and re-infection and relapse rate estimates provided in the appendix (p 12). Numbers at risk are in the appendix (p 9). PvRM=*Plasmodium vivax* Recurrence Model.

Median hypnozoiticidal efficacies are shown in table 2. Estimates for the two low-dose primaquine regimens (3·5 mg/kg total), administered over 7 and 14 days, were the same because we only had access to pooled data for these regimens.^11^ CrIs for the tafenoquine regimens were quite wide owing to the scarce clinical trial data on tafenoquine regimens (appendix p 3).^12^ The PvRM produces simulations that are consistent with clinical trial data (figure 1; appendix pp 10–11). In the sensitivity analyses, efficacy estimates were stable with respect to variations in the values of fixed parameters, when modelling an alternative mechanism of hypnozoite elimination, and when fitting to a subset of clinical trial data (appendix pp 13–14).

**Table 2:** Estimates of per-patient hypnozoiticidal efficacy and effectiveness for various 8-aminoquinoline regimens

	Median hypnozoiticidal efficacy*(95% CrI)	**Median hypnozoiticidal effectiveness under trial conditions**†**(95% CrI)**	**Median hypnozoiticidal effectiveness under operational conditions**‡**(95% CrI)**
No 8-aminoquinolines	0% (0–0)§	0% (0–0)	0% (0–0)
Primaquine (3·5 mg/kg over 14 days)	72·3% (68·1–76·3)¶	68·7% (64·6–72·5)‖	36·0% (33·9–38·0)
Primaquine (3·5 mg/kg over 7 days)	72·3% (68·1–76·3)¶	68·7% (64·6–72·5)‖	42·4% (39·9–44·7)
Primaquine (7 mg/kg over 14 days)	99·1% (96·0–100)**	94·2% (91·2–95·0)‖	49·4% (47·9–49·8)
Primaquine (7 mg/kg over 7 days)	96·3% (90·8–99·7)**	91·5% (86·2–94·7)‖	54·7% (51·5–56·7)††
Tafenoquine (5 mg/kg single dose)	62·4% (49·1–76·3)‡‡	62·4% (49·1–76·3)	54·6% (44·2–65·5)††
Tafenoquine (7·5 mg/kg single dose)	87·5% (62·1–99·3)‡‡	87·5% (62·1–99·3)	73·5% (54·0–82·8)††
Magic bullet (single dose)	100% (100–100)§	100% (100–100)	100% (100–100)

The first column summarises the posterior distributions of the hypnozoiticidal efficacy estimates generated under the *Plasmodium vivax* Recurrence Model with the all-or-nothing mechanism. The second and third columns show estimates for the hypnozoiticidal effectiveness under trial conditions and operational conditions. Parameter values used to produce this table are listed in the appendix (pp 6, 18–20). CrI=credible interval. CYP2D6=cytochrome P450 2D6. G6PD=glucose-6-phosphate dehydrogenase.

* Denominator is patients who are eligible for the 8-aminoquinoline regimen, perfectly adhere, and are CYP2D6 normal.

† Denominator is patients who are eligible for the 8-aminoquinoline regimen and who perfectly adhere.

‡ Denominator is patients who seek treatment.

§ Assumption.

¶ Fitted to meta-analysis of patient-level trial data.^11^

‖ Patients who are low CYP2D6 metabolisers are unable to process primaquine.

** Fitted to patient-level clinical trial data.^9^

†† Alternative 8-aminoquinoline regimen: patients who are not eligible for the use of tafenoquine or primaquine (7 mg/kg over 7 days) are prescribed primaquine (3·5 mg/kg over 7 days) provided they have a G6PD activity greater than 30%, are not pregnant or breastfeeding, and are aged 6 months or older.

‡‡ Fitted to meta-analysis of patient-level trial data.^12^

Under trial conditions, the mean per-patient hypnozoiticidal effectiveness of primaquine regimens was reduced in proportion to the prevalence of individuals with low CYP2D6 metabolism (table 2). We estimated a mean per-patient hypnozoiticidal effectiveness under operational conditions of 36·0% (95% CrI 33·9–38·0) for 14-day low-dose primaquine, 42·4% (39·9–44·7) for 7-day low-dose primaquine, 49·4% (47·9–49·8) for 14-day high-dose primaquine, 54·7% (51·5–56·7) for 7-day high-dose primaquine, 54·6% (44·2–65·5) for low-dose tafenoquine, and 73·5% (54·0–82·8) for high-dose tafenoquine (table 2).

**Figure 2:**
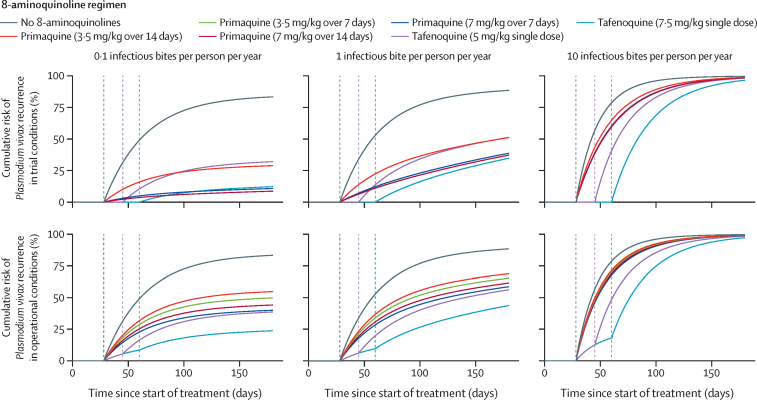
Cumulative risk of *Plasmodium vivax* recurrence after treatment of primary blood-stage infection Trial conditions denotes a hypothetical cohort of trial participants in which all patients are eligible for the selected 8-aminoquinoline regimen and drug administration is supervised by a trained clinician. Operational conditions denotes a group representative of real-world patients for whom eligibility for primaquine and tafenoquine is restricted and where we assumed 67% adherence to 7-day primaquine regimens and 57% adherence to 14-day primaquine regimens (appendix p 18). We used the *Plasmodium vivax* Recurrence Model to produce this figure. Vertical dashed lines represent the end of the post-treatment prophylaxis period (appendix pp 18–19) for tafenoquine at 7·5 mg/kg (light blue), tafenoquine at 5 mg/kg (purple), and all other drug regimens (grey). All patients are treated with chloroquine for blood-stage activity.

The risk of recurrence over time in patients enrolled into a hypothetical clinical trial and a group representative of real-world patients is shown in figure 2. Under trial conditions, differences in the risk of *P vivax* recurrence vary based on the duration of post-treatment prophylaxis (a longer prophylaxis period corresponds to a later onset of recurrence risk) and the hypnozoiticidal efficacy of the 8-aminoquinoline component (a higher efficacy corresponds to a slower increase of recurrence risk over time). Under operational conditions, primaquine regimens are not adhered to perfectly (explaining the faster increase of recurrence risk over time) and some patients who are ineligible for regimens using tafenoquine or primaquine at 7 mg/kg over 7 days will instead be given primaquine at 3·5 mg/kg over 7 days (patients given primaquine start to recur earlier than those given tafenoquine).

**Figure 3:**
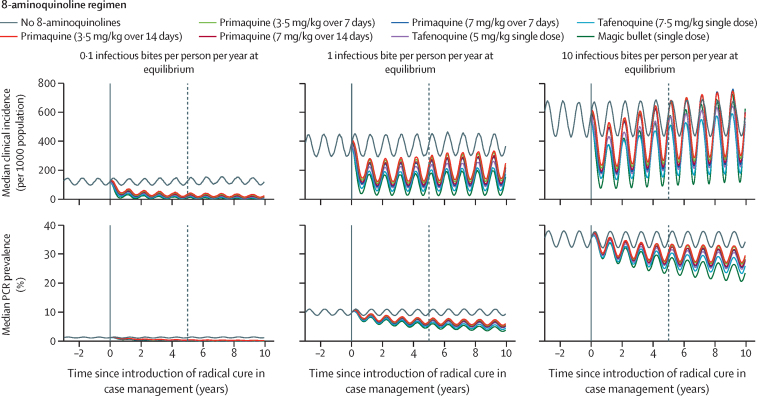
Simulated *Plasmodium vivax* transmission in a community with moderate malaria seasonality Radical cure is introduced in year 0 (solid vertical lines) and its effect is evaluated in year 5 (dashed vertical lines). Clinical incidence and prevalence as measured by PCR are the medians of 100 independent simulations per scenario using the *Plasmodium vivax* Individual-Based Model. All patients were assumed to be treated with chloroquine for blood-stage activity.

In low-transmission settings, prevalence as measured by PCR and clinical incidence decreased following the introduction of radical cure in case management (figure 3). In low transmission settings, the intervention reduces both relapses and re-infections (appendix p 23). In areas where transmission is greater, prevalence as measured by PCR decreases slowly but steadily; conversely, initial decreases in clinical incidence are followed by a rebound in cases (figure 3). In the first 2 years, relapses strongly decrease but then start to slowly increase again; conversely, re-infections start increasing from the first year of the intervention. Introducing radical cure case management is estimated to prevent 365–509 cases per 1000 population over 5 years in areas where pre-intervention *P vivax* transmission was low, and 679–1404 cases per 1000 population over 5 years where pre-intervention transmission was high (appendix p 24).

**Figure 4:**
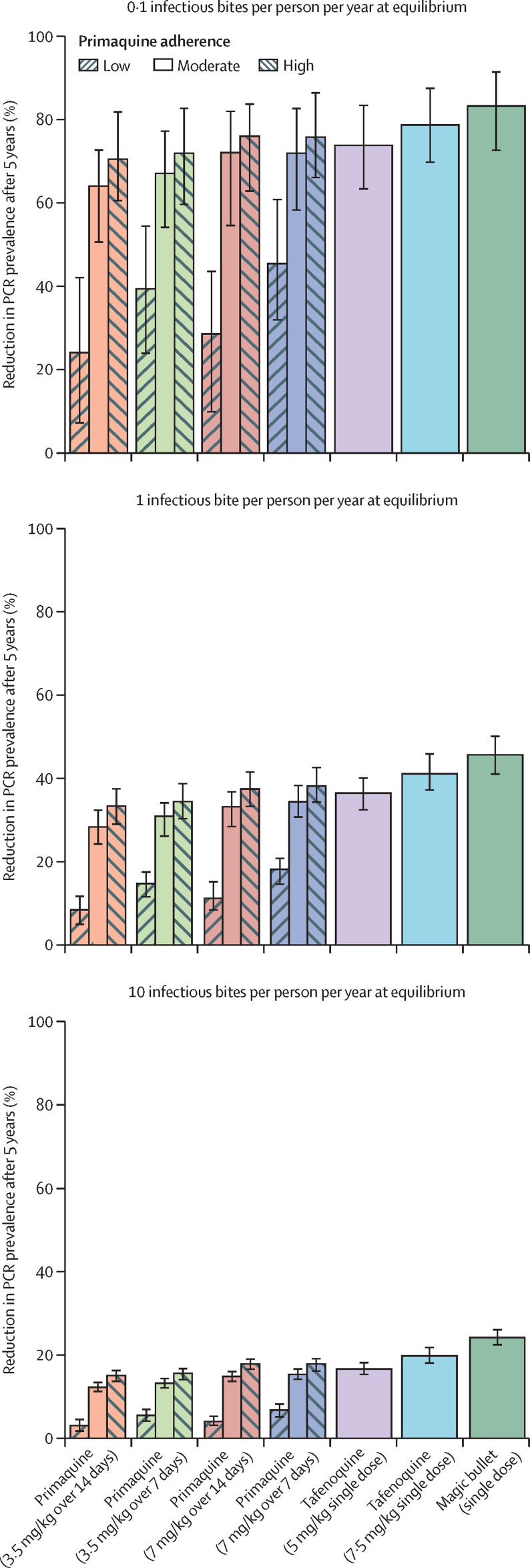
Effect of introducing radical cure on *Plasmodium vivax* prevalence as measured by PCR Effect is reported as the reduction in prevalence as measured by PCR after 5 years from introducing radical cure in *Plasmodium vivax* case management. We report the medians and 95% CIs computed from 100 independent simulations per scenario using the *Plasmodium vivax* Individual-Based Model. All patients were assumed to be treated with chloroquine for blood-stage activity.

Introducing a hypothetical magic bullet could reduce prevalence as measured by PCR by a median of 83% (95% CI 73–92) over 5 years in low-transmission settings and by a median of 24% (22–26) in high-transmission settings (figure 4). These quantities represent the theoretical maximum impact that an 8-aminoquinoline regimen could achieve. Tafenoquine regimens were estimated to achieve reductions in prevalence as measured by PCR over 5 years of 74–79% where pre-intervention transmission was low (<2%) and 17–20% where pre-intervention transmission was high (around 35%). Assuming primaquine adherence is moderate (67% for 7-day regimens and 57% for 14-day regimens) or high (90% for 7-day regimens and 80% for 14-day regimens), primaquine regimens are estimated to achieve reductions in prevalence as measured by PCR over 5 years similar to tafenoquine: 64–76% in low-transmission settings and 12–18% in high-transmission settings. Conversely, assuming primaquine adherence is poor (20% for 7-day regimens and 10% for 14-day regimens), the prevalence reduction is smaller over 5 years: 24–45% in low-transmission settings and 3–7% in high-transmission settings. With the exception of pre-intervention transmission intensity and the level of primaquine adherence, estimates of reduction in prevalence as measured by PCR were stable with respect to different levels of case management coverage (ie, the proportion of febrile patients receiving treatment), transmission setting characteristics, and when assuming that relapses elicit a weaker immune response than primary blood-stage infections (appendix pp 26–28).

## Discussion

An accurate evaluation of the efficacy of 8-aminoquinoline regimens is necessary for their optimal implementation in *P vivax* control and elimination interventions. To our knowledge, our modelling framework is the first to generate hypnozoiticidal efficacy estimates for a broad range of 8-aminoquinoline regimens from prospective clinical trial data. 8-aminoquinoline regimens using high-dose primaquine (7 mg/kg total) are estimated to have an almost perfect hypnozoiticidal efficacy, followed by high-dose tafenoquine (7·5 mg/kg total), low-dose primaquine (3·5 mg/kg total), and low-dose tafenoquine (5 mg/kg total). Introducing radical cure case management in low-transmission settings has the potential to reduce transmission to pre-elimination levels. In high-transmission settings, radical cure case management is predicted to have a moderate impact on *P vivax* control.

In line with previous studies,^11^, ^12^ we found that hypnozoiticidal efficacy increases with 8-aminoquinoline dose. Our estimates for the hypnozoiticidal efficacy of 7 mg/kg of primaquine over 7 or 14 days are consistent with that produced by Taylor and colleagues^3^ using genotyping data from different trials. Adekunle and colleagues^16^ reported a notably lower hypnozoiticidal efficacy of 7 mg/kg of primaquine over 14 days using time-to-infection data from a clinical trial in children from Papua New Guinea. We believe this difference is due to the combination of primaquine with 7 days of artesunate monotherapy in this study,^17^ in contrast to the more routinely used chloroquine or artemisinin-based combination therapy. Our results add to the mounting evidence that clinical trials underestimate the hypnozoiticidal efficacy of 8-aminoquinolines.^3^, ^18^ Our modelling approach to estimate hypnozoiticidal efficacy could be applied to ongoing clinical trials of 8-aminoquinoline regimens.

Our analysis provides new insights into the patient-level and community-level impact of radical cure. High theoretical efficacy estimates translate to notably lower estimates of per-patient effectiveness due to imperfect drug adherence (primarily penalising primaquine regimens^19^, ^20^) and 8-aminoquinoline eligibility restrictions (primarily penalising tafenoquine regimens^21^). The same factors also limit the community-level impact of radical cure case management. Even though high-dose primaquine (7 mg/kg total) was estimated to have the highest hypnozoiticidal efficacy, it achieves suboptimal reductions in community transmission when adherence is less than 50%.

The PvRM made several simplifying assumptions. We assumed that all patients remain in the study site and continue to be exposed to new infections from mosquito bites for the duration of follow-up, potentially leading to further blood-stage and liver-stage infections. Relapse^22^ and infection rates were assumed to be location-specific, but drug efficacy was assumed to remain constant across locations. In particular, we assumed that relapse rates,^23^ hypnozoiticidal efficacy,^18^, ^24^ and hypnozoite clearance^22^ do not depend on the patient's hypnozoite load, even though we know this not to be the case. For instance, as *P vivax* incidence decreases, hypnozoite loads will decrease and hypnozoiticidal efficacy might improve.^25^ Knowledge of trial participants' history of previous *P vivax* infections would have allowed us to make better informed assumptions on their immunity levels and their hypnozoite burden. Moreover, we assumed that all trial patients carried hypnozoites at enrolment, thereby biasing hypnozoiticidal efficacy estimates upwards. Conversely, excluding the possibility of schizontocidal treatment failure (and thus ignoring recrudescence) might bias hypnozoiticidal efficacy estimates downwards.

Among the clinical trial data used to calibrate the PvRM, all tafenoquine^12^ and more than 99% of high-dose primaquine (7 mg/kg total) treatment courses^9^, ^11^ were fully supervised; however, only a third of low-dose primaquine (3·5 mg/kg total)^12^ treatment courses were fully supervised and the rest partially supervised. Our modelling assumption of perfect 8-aminoquinoline adherence might thus have biased downwards our hypnozoiticidal efficacy estimates for low-dose primaquine (3·5 mg/kg total). Even though artemisinin-based combination therapy has been linked with reduced tafenoquine efficacy^10^ (and suspected to reduce primaquine efficacy), we assumed hypnozoiticidal efficacy to be independent of the blood-stage drug because chloroquine was by far the most widely used partner drug. Last, we assumed all patients to have the same probability of being low CYP2D6 metabolisers.

We made several assumptions when simulating community-level transmission using the PvIBM. We assumed chloroquine to be the only prescribed schizontocidal drug and excluded the possibility of blood-stage treatment failures. This might bias upwards our estimates for the prevalence reduction of radical cure case management. In the PvIBM, patients who are prescribed primaquine either fully adhere to this regimen, taking all the doses at the appropriate time, or do not take a single primaquine dose. In reality, adherence is likely to be more heterogeneous, with patients taking varying proportions of the prescribed dosage.^19^ Dosing 8-aminoquinolines based on a patient's weight is usually feasible in clinical trials; however, weight banding is commonly used under operational conditions, leading to imprecise dosing. Finally, we did not simulate haemolytic events caused by incorrect G6PD testing (either due to misclassification by the instrument or human error) due to a scarcity of reliable data on the frequency of such events. Since the risk of severe haemolysis was not quantified, we were unable to consider a broader analysis of the relative risks and benefits of 8-aminoquinoline treatment.^8^

Current WHO guidelines on preventing relapses recommend administering schizontocidal drugs in combination with high-dose primaquine (7 mg/kg total) given over 14 days in areas with fast relapsing *P vivax* strains (ie, east Asia and Oceania) or with low-dose primaquine (3·5 mg/kg total) given over 7 or 14 days elsewhere.^26^ To mitigate the risk of haemolysis, these primaquine regimens should only be prescribed to patients with more than 30% G6PD activity.^26^ However, routine testing of G6PD activity is rarely implemented,^1^ and thus some countries prescribe low-dose primaquine instead of the recommended high-dose primaquine.^4^ A meta-analysis showed the safety of high-dose primaquine (7 mg/kg total) administered over 7 days to patients with more than 70% G6PD activity.^27^

WHO is currently reviewing the use of tafenoquine for the prevention of *P vivax* relapses.^26^ However, low-dose tafenoquine (5 mg/kg single dose), when administered in combination with chloroquine, is licensed for patients with more than 70% G6PD activity in several countries.^10^ A paediatric formulation of low-dose tafenoquine has also recently been marketed for children aged 2 or older. The efficacy of high-dose tafenoquine (7·5 mg/kg single dose) was explored in a recent meta-analysis;^12^ however, the safety and tolerability profile of this regimen needs to be examined in prospective clinical trials.^28^

In symptomatic patients, high-dose radical cure is warranted if G6PD deficiency can be excluded; this approach will reduce the number of recurrent infections and improve associated health outcomes.^7^, ^11^, ^12^ From a public health perspective, both low-dose and high-dose primaquine and tafenoquine regimens will cause reductions in transmission. However, in situations where radical cure is not currently implemented, and where there are safety concerns related to high-dose regimens, a pragmatic approach is to opt for case management with low-dose regimens. Such a strategy would probably reduce *P vivax* transmission substantially, while health facilities gain operational experience prescribing 8-aminoquinolines and testing for G6PD deficiency. A switch to high-dose regimens and a further push towards *P vivax* elimination could then occur after transmission has been reduced (resulting in fewer treatments prescribed) and in a setting with more experienced health systems (resulting in safer patient outcomes).

To maximise the impact of radical cure case management, it is essential to provide safe drug treatment via the systematic use of G6PD testing, selecting optimal doses for primaquine and tafenoquine, and improving adherence and coverage. Our hypnozoiticidal efficacy estimates allow mathematical modellers and health economists to parametrise further analyses on the impact^13^, ^14^ and cost-effectiveness^29^, ^30^ of various radical cure interventions.

### Contributors

### Data sharing

The code used for this study will be made publicly available with publication at https://github.com/ConniCia/radical_cure. De-identified data on the time to first *P vivax* recurrence from the IMPROV clinical trial was obtained following instructions described by Taylor and colleagues.^9^ All other relevant data were obtained from publicly available sources and are provided in the appendix.

## Declaration of interests

We declare no competing interests.
