# Supplementary material for: Quantifying Plasmodium vivax radical cure efficacy: a modelling study integrating clinical trial data and transmission dynamics
Source: Lancet Infect Dis. 2025 Jun;25(6):668–77. doi: 10.1016/S1473-3099(24)00689-3 (PMC12095116; doi:10.1016/S1473-3099(24)00689-3)
Supplement: Supplementary appendix [file mmc1.pdf]

THE LANCET
Infectious Diseases

Supplementary appendix

This appendix formed part of the original submission and has been peer reviewed. We post it as supplied by the authors.

Supplement to: Ciavarella C, Drakeley C, Price RN, Mueller I, White M. Quantifying *Plasmodium vivax* radical cure efficacy: a modelling study integrating clinical trial data and transmission dynamics. *Lancet Infect Dis* 2025; published online Jan 13. https://doi.org/10.1016/S1473-3099(24)00689-3.

THE LANCET
Infectious Diseases

Supplementary appendix

This appendix formed part of the original submission and has been peer reviewed. We post it as supplied by the authors.

Supplement to: Ciavarella C, Drakeley C, Price RN, Mueller I, White M. Quantifying *Plasmodium vivax* radical cure efficacy: a modelling study integrating clinical trial data and transmission dynamics. *Lancet Infect Dis* 2025; published online Jan 13. https://doi.org/10.1016/S1473-3099(24)00689-3.

Quantifying *P. vivax* radical cure efficacy: a modelling study integrating clinical trial data and transmission dynamics

Supplementary Information

Constanze Ciavarella[1], Chris Drakeley[2], Ric N Price[3,4,5], Ivo Mueller[6], and Michael White[1,]

[1] Institut Pasteur, Université Paris Cité, G5 Épidémiologie et Analyse des Maladies Infectieuses, Paris, France
[2] London School of Hygiene and Tropical Medicine, London, UK
[3] Global and Tropical Health Division, Menzies School of Health Research and Charles Darwin University, Darwin, NT 0810, Australia
[4] Centre for Tropical Medicine and Global Health, Nuffield Department of Medicine, University of Oxford, Oxford, UK
[5] Mahidol-Oxford Tropical Medicine Research Unit, Faculty of Tropical Medicine, Mahidol University, Bangkok, Thailand
[6] Walter and Eliza Hall Institute, Parkville, VIC, Australia

Correspondence: Michael White <michael.white@pasteur.fr>

Table of contents

Overview of modelling algorithm 2
Estimating hypnozoiticidal efficacy from prospective clinical trial data using the *P. vivax* Recurrence Model (PvRM) .. 3
- Clinical trial data 3
- Model description 4
- Mathematical formulation 4
- Closed form analytical solution 5
- Statistical inference framework 6
- Log-likelihood function for the IMPROV trial 7
- Log-likelihood function for the Commons et al meta-analysis 9
- Log-likelihood function for the Watson et al meta-analysis 10
- Additional results 12
- Sensitivity analyses 13

Extending the PvRM to operational conditions 15
- Compute the proportion of a population eligible for each 8-aminoquinoline regimen 15
- Computing the cumulative incidence of blood-stage recurrence under operational conditions 16

Quantifying the community-level impact of radical cure case management using the *P. vivax* Individual-Based Model (PvIBM) 17
- Model overview 17
- Model parameters 18
- Additional results 21
- Sensitivity analyses 26

References 29

Overview of modelling algorithm

- Estimate the hypnozoiticidal efficacy of various primaquine (PQ) and tafenoquine (TQ) regimens using the *P. vivax* Recurrence Model (PvRM).
  - Estimate the hypnozoiticidal efficacy of 7 mg/kg of PQ over 7 and 14 days by fitting (Equation 5) to clinical trial data from the IMPROV study.[1]
  - Fix the hypnozoiticidal efficacy of 7 mg/kg of PQ to the median estimate from the previous step, then estimate the hypnozoiticidal efficacy of 3·5 mg/kg of PQ by fitting (Equation 6) to data from a meta-analysis of clinical trials.[2]
  - Fix the hypnozoiticidal efficacy of 3·5 mg/kg of PQ to the median estimate from the previous step, then estimate the hypnozoiticidal efficacy of 5 and 7·5 mg/kg of TQ by fitting (Equation 7) to data from a meta-analysis of clinical trials.[3]
- Quantify the patient- and community-level impact of radical cure case management under operational conditions.
  - Fix the hypnozoiticidal efficacies of 8-aminoquinoline (8-AQ) regimens to the median estimates from the previous steps.
  - Use the PvRM extension accounting for 8-AQ eligibility and adherence to quantify the patient-level impact.
  - Use the existing *P. vivax* Individual-Based Model (PvIBM) to quantify the community-level impact.

Estimating hypnozoiticidal efficacy from prospective clinical trial data using the *P. vivax* Recurrence Model (PvRM)

Clinical trial data

We accessed publicly available data from a recent prospective clinical trial[1] and two meta-analyses of clinical trials[2,3] (Table S1).

	IMPROV trial[1]	Meta-analysis[2]	Meta-analysis[3]
PQ supervised	Fully (100%)	Fully (63·4%), partially (33·5%)	Fully (32·2%), partially (67·8%)
Partner blood-stage drug	CQ, except DP in Indonesia	CQ (67·1%), DP (23·1%), other ACT (9·8%)	CQ
Follow-up period	365 days	Varied, minimum 42 days	180 days
Recurrence endpoint	First symptomatic *P. vivax* recurrence	First *P. vivax* recurrence	First *P. vivax* recurrence
Locations	Afghanistan, Ethiopia, Indonesia, Vietnam	Asia-Pacific, Americas, Africa	Asia-Pacific, Americas, Africa
Trial arms	Placebo (n=472), PQ 7 mg/kg over 7 days (n=942), PQ 7 mg/kg over 14 days (n=945)	Control* (n=1470), PQ 3·5 mg/kg (n=2569), PQ 7 mg/kg (n=2811)	Placebo (n=182), PQ 3·5 mg/kg over 14 days (n=257), TQ 5 mg/kg single dose (n=368), TQ 7·5 mg/kg single dose (n=54)

Table S1: Overview of clinical trial data used to fit the P. vivax Recurrence Model (PvRM). (8-AQ) 8-aminoquinoline, (ACT) artemisinin-based combination therapy, (CQ) chloroquine, (DP) dihydroartemisinin-piperaquine, (PQ) primaquine, (TQ) tafenoquine. * Control refers to placebo or no 8-AQ given.

First, the risks of recurrence following different treatment regimens were derived from Kaplan-Meier site estimates in the IMPROV randomised, placebo-controlled, double-blind clinical trial.[1] The trial recruited patients with >30% G6PD activity in eight locations across Afghanistan, Ethiopia, Indonesia, and Vietnam. All patients were treated with schizontocidal drugs (chloroquine or dihydroartemisinin-piperaquine) and assigned randomly to treatment with one of three trial regimens: 7 mg/kg of PQ over 7 days (942 patients), 7 mg/kg of PQ over 14 days (945 patients), or placebo (472 patients). For each arm, the time of first symptomatic blood-stage recurrence or the time of drop-out is known for a period of 365 days.

The meta-analysis by Commons et al[2] included patients with >30% G6PD activity divided into three groups (Table S2): 3·5 mg/kg of PQ (2569 patients), 7 mg/kg of PQ (2811 patients), and control (1470 patients). All patients were treated with schizontocidal drugs. For each group, the meta-analysis specified the Kaplan-Meier estimate for the risk of blood-stage recurrence by day 180. The data are aggregated across multiple locations.

8-AQ regimen	Nr enrolled	Proportion recurred by day 180 (%)
No 8-AQs	1470	51 [48·2, 53·9]
PQ (3·5 mg/kg)	2569	19·3 [16·9, 21·9]
PQ (7 mg/kg)	2811	8·1 [7, 9·4]

Table S2: Data from meta-analysis[2] used to estimate the hypnozoiticidal efficacy of low-dose PQ regimens (3·5 mg/kg total). (8-AQ) 8-aminoquinoline, (PQ) primaquine.

The meta-analysis of placebo-controlled clinical trials by Watson et al[3] included patients with >70% G6PD activity divided into four groups (Table S3): 3·5 mg/kg of PQ over 14 days (257 patients), 5 mg/kg of TQ (368 patients), 7·5 mg/kg of TQ (54 patients), and placebo (182 patients). All patients were treated with CQ. For each group, the meta-analysis presented the number of patients with a blood-stage recurrence by day 120. At this time only 1·1% of patients were lost to follow up and thus drop-outs were ignored. The data were aggregated across multiple locations.

8-AQ regimen	Nr enrolled	Nr recurred by day 120
No 8-AQs	182	101
PQ (3·5 mg/kg over 14 days)	257	57
TQ (5 mg/kg single dose)	368	79
TQ (7·5 mg/kg single dose)	54	4

Table S3: Data from meta-analysis[3] used to estimate the hypnozoiticidal efficacy of TQ regimens. (8-AQ) 8-aminoquinoline, (PQ) primaquine, (TQ) tafenoquine.

Model description

The PvRM describes *P. vivax* recurrence in patients that have been treated for symptomatic *P. vivax*. Consider a *P. vivax*-endemic region where patients get infected through mosquito bites at a constant rate λ. Patients that carry liver-stage parasites (hypnozoites) additionally relapse at a constant rate f, and clear hypnozoites at a constant rate γ.

At day $t = 0$, assume to observe a group of patients seeking treatment for an ongoing symptomatic blood-stage infection. We assume each of these patients to also carry hypnozoites. All patients immediately start the same drug regimen consisting either of a schizontocidal drug alone, or a schizontocidal drug in combination with a hypnozoiticidal drug (radical cure). The schizontocidal drug is assumed to clear all blood-stage parasites (i.e. we exclude recrudescences), but keep hypnozoites intact. If the drug regimen includes a liver-stage drug, all patients also take a full course of an 8-AQ that acts on their liver-stage infection.

We model two distinct mechanisms of hypnozoite elimination.[4] In the "all-or-nothing" mechanism, 8-AQs were assumed to eliminate all hypnozoites in a fraction ε of patients, while the remaining fraction $1 - \varepsilon$ of patients will still carry their original amount of hypnozoites. In the "leaky" mechanism, 8-AQs were assumed to eliminate a fraction ε of hypnozoites in all patients, such that they relapse at a slower rate of $(1 - \varepsilon) \cdot f$.

We assume the drug regimen to provide post-treatment prophylaxis for a number d of days following treatment thereby protecting patients from blood-stage recurrences. Moreover, we assume PQ to fail in a fraction, c of patients who are low cytochrome P450 2D6 (CYP2D6) metabolisers[5–7]. Note that TQ is not affected by lower CYP2D6 metabolisation[6].

Mathematical formulation

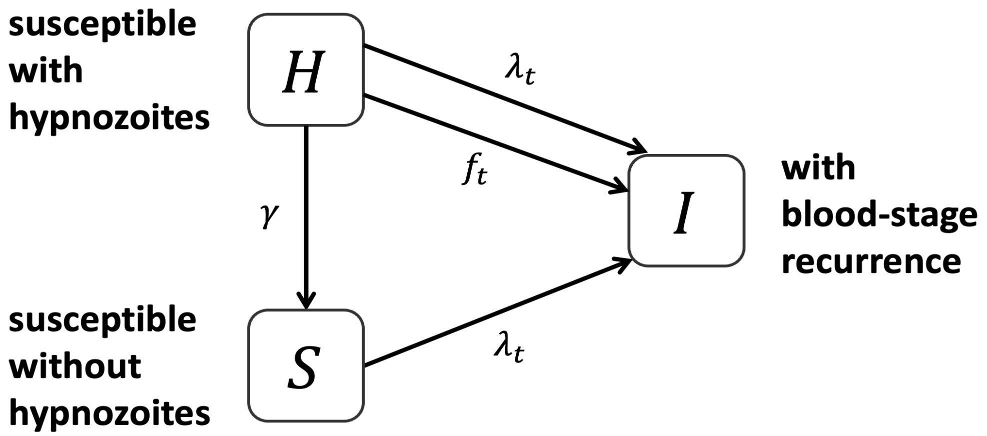

Figure S1: Compartmental representation of the P. vivax Recurrence Model (PvRM). The PvRM describes the recurrence of P. vivax blood-stage infections in patients that have been treated for symptomatic P. vivax at time $t = 0$. Susceptible patients that carry hypnozoites (compartment H) relapse at a rate f_t, get reinfected at a rate λ_t, and clear hypnozoites without having a recurrence at a rate γ. Susceptible patients without hypnozoites (compartment S) get reinfected at a rate λ_t. Patients that have had a blood-stage recurrence after drug treatment are represented by compartment I.

The PvRM described above can be formulated as a deterministic compartmental model (Figure S1). The parameters introduced above are

$$\begin{aligned}
\lambda &= \text{daily reinfection rate},\\
f &= \text{daily relapse rate},\\
d &= \text{post-treatment prophylaxis period with no blood-stage recurrences (days)},\\
\varepsilon &= \text{hypnozoiticidal efficacy in normal CYP2D6 metabolisers (\%)},\\
c &= \text{fraction of patients who are low CYP2D6 metabolisers (\%)},\\
\gamma &= \text{rate of hypnozoite clearance}.
\end{aligned}$$

Note that d is defined as the maximum post-treatment prophylaxis duration of the administered antimalarial drugs. Let $t \geq 0$ denote time in days, and $t = 0$ denote the day of drug treatment onset. We define the following state variables

$$\begin{aligned}
H(t) &= \text{fraction of susceptible patients with hypnozoites at time } t \geq 0,\\
S(t) &= \text{fraction of susceptible patients without hypnozoites at time } t \geq 0,\\
I(t) &= \text{fraction of patients who have had a blood-stage recurrence by time } t \geq 0,
\end{aligned}$$

where $H(t) + S(t) + I(t) = 1$ for all $t \geq 0$.

Introducing the following variables

$$\begin{aligned}
\varepsilon_c &= \begin{cases} 0 & \text{if no 8-AQ is employed} \\ \varepsilon & \text{if TQ is employed} \\ \varepsilon(1-c) & \text{if PQ is employed}, \end{cases}\\
f_m &= \begin{cases} f & \text{if mechanism of hypnozoite elimination = all-or-nothing} \\ f(1-\varepsilon_c) & \text{if mechanism of hypnozoite elimination = leaky}, \end{cases}\\
f_t &= \begin{cases} 0 & \text{if } t < d \\ f_m & \text{if } t \geq d, \end{cases}\\
\lambda_t &= \begin{cases} 0 & \text{if } t < d \\ \lambda & \text{if } t \geq d, \end{cases}
\end{aligned}$$

we can formulate the PvRM using the following system of ordinary differential equations

$$\begin{cases} H'(t) &= -(\lambda_t + f_t + \gamma)H, \\ S'(t) &= \gamma H - \lambda_t S, \\ I'(t) &= (\lambda_t + f_t)H + \lambda_t S. \end{cases} \qquad (3)$$

Since we account for post-treatment prophylaxis, we can make the simplifying assumption that antimalarial drugs act immediately. Thus, the initial conditions on day $t = 0$ will be

$$\begin{cases} h_0 & := H(0) = \begin{cases} 1-\varepsilon_c & \text{if mechanism of hypnozoite elimination = all-or-nothing} \\ 1 & \text{if mechanism of hypnozoite elimination = leaky}, \end{cases} \\ s_0 & := S(0) = \begin{cases} \varepsilon_c & \text{if mechanism of hypnozoite elimination = all-or-nothing} \\ 0 & \text{if mechanism of hypnozoite elimination = leaky}, \end{cases} \\ i_0 & := I(0) = 0. \end{cases} \qquad (4)$$

Closed form analytical solution

The system of equations (Equation 3) with initial conditions (Equation 4) can be solved analytically in two steps. For $t < d$ the ODE system simplifies to

$$\begin{cases} H'(t) &= -\gamma H, \\ S'(t) &= \gamma H, \\ I'(t) &= 0, \end{cases}$$

and its analytical solution is

$$\begin{cases} H(t) &= h_0 e^{-\gamma t}\,, \\ S(t) &= 1 - h_0 e^{-\gamma t}\,, \\ I(t) &= 0\,. \end{cases}$$

For $t \geq d$ the system of equations becomes

$$\begin{cases} H'(t) &= -(\lambda + f_m + \gamma)H\,, \\ S'(t) &= \gamma H - \lambda S\,, \\ I'(t) &= (\lambda + f_m)H + \lambda S\,, \end{cases}$$

with new initial conditions

$$\begin{cases} h_d &:= H(d) = h_0 e^{-\gamma d}\,, \\ s_d &:= S(d) = 1 - h_0 e^{-\gamma d}\,, \\ i_d &:= I(d) = 0\,. \end{cases}$$

The analytical solution for $t \geq d$ is

$$\begin{cases} H(t) &= h_d e^{-(\gamma+\lambda+f_m)(t-d)}\,, \\ S(t) &= \dfrac{1}{f_m+\gamma}\left[(f_m s_d + \gamma)e^{-\lambda(t-d)} - \gamma h_d e^{-(\gamma+\lambda+f_m)(t-d)}\right], \\ I(t) &= 1 - \dfrac{1}{f_m+\gamma}\left[(f_m s_d + \gamma)e^{-\lambda(t-d)} + f_m h_d e^{-(\gamma+\lambda+f_m)(t-d)}\right]. \end{cases}$$

Statistical inference framework

In this section, we describe how we use the PvRM to estimate hypnozoiticidal efficacy from prospective clinical trial data using Gibbs sampling, a Bayesian Markov Chain Monte Carlo (MCMC) method. Parameters c, γ, and d are derived from the literature and will be varied in sensitivity analyses (Table S4). The duration, d, of post-treatment prophylaxis against recurrent infections of a specific 8-AQ drug regimen is calculated as the maximum of the post-treatment prophylaxis duration of its component drugs. Note that the post-treatment prophylaxis of PQ (Table S8) is always shorter than that of schizontocidal drugs. We will use the all-or-nothing mechanism of hypnozoite elimination in the main analysis, and the leaky mechanism in sensitivity analyses.

Description	Parameter	Default value	Alternative values	Reference for default value
Prevalence of CYP2D6 low metabolisers	c	0·05	0, 0·1	[8]
Mean duration of hypnozoite carriage (in days)	$1/\gamma$	383	283, 483	[9]
Duration of CQ prophylaxis (in days)	d	28	14, 42	[10]
Duration of DP prophylaxis (in days)	d	42	28, 56	[10]
Duration of TQ 5 mg/kg prophylaxis (in days)	d	45	31, 59	[11]
Duration of TQ 7·5 mg/kg prophylaxis (in days)	d	60	46, 74	Assumption

Table S4: Default and alternative parameter values for the P. vivax Recurrence Model (PvRM). (CQ) chloroquine, (DP) dihydroartemisinin-piperaquine, (TQ) tafenoquine.

Parameters λ, f, and ε are unknown quantities that need to be determined by fitting the PvRM to clinical trial data. Note that since the PvRM is a compartmental model, we implicitly assume λ and f to be exponentially distributed. The trial data summarised above only provides information on blood-stage recurrences and loss to trial follow-up. It is thus sufficient to fit the functional form of the cumulative incidence of blood-stage recurrences over time $I(t)$ to the data

$$I(t \,|\, m, c, \gamma, d, \lambda, f, \varepsilon) = \begin{cases} 0 & \text{if } t < d \\ 1 - \dfrac{1}{f_m+\gamma}\left[(f_m s_d + \gamma)e^{-\lambda(t-d)} + f_m h_d e^{-(\gamma+\lambda+f_m)(t-d)}\right] & \text{if } t \geq d\,, \end{cases}$$

where we have denoted all parameter dependences and m denotes the mechanism of hypnozoite elimination. For simplicity of notation, we will often write the cumulative incidence as only depending on unknown parameters

$$I(t) = I(t \,|\, \lambda, f, \varepsilon)\,.$$

We assume infection rates λ and relapse rates f to be location-specific, and hypnozoiticidal efficacies ε to solely depend on the 8-AQ regimen. Adherence to radical cure regimens is assumed to be 100% under trial conditions. Recall that hypnozoiticidal efficacy of schizontocidal drugs is assumed to be 0%. We assume uniform priors for each unknown parameter

$$\begin{aligned} \varepsilon &\sim \text{Uniform}(0,\, 1)\,, \\ \lambda, f &\sim \text{Uniform}\left(0,\, \frac{15}{365}\right)\,. \end{aligned}$$

Since probability distributions on uniform priors return a constant value, we ignore the contribution of the prior on the log-likelihoods defined in the next section. We fitted the PvRM separately to each of the three clinical trial datasets (Table S1) and therefore define three distinct log-likelihood function (see subsequent sections). For each such fit, we performed Latin hypercube sampling to identify suitable starting conditions for 6 MCMC chains generated using Gibbs sampling. Each of these chains was run for 50,000 iterations, thinning per 10 iterations, and discarding 25% for burn-in. Convergence of MCMC chains was assessed visually and calculating the multivariate effective sample size.[12]

Log-likelihood function for the IMPROV trial

To estimate the hypnozoiticidal efficacy of 7 mg/kg of PQ over 7 and 14 days, we used patient-level data from the IMPROV trial.[1] Let $G_{x,y}$ be the set of patients of location x and treatment arm y. For each patient $i \in G_{x,y}$, we have information on their time to event t_i, and whether the event denotes first symptomatic blood-stage recurrence $j_i = 1$ or trial drop-out $j_i = 0$. We exclude all patients i for which $t_i < d$.

Note that $I(t)$ is a cumulative distribution function since (1) it is non-decreasing, (2) it is right-continuous, (3) is has support on the closed interval $[0,1]$, and (4) it satisfies $\lim_{t\to 0} I(t) = 0$ and $\lim_{t\to\infty} I(t) = 1$.

Let T_i be a random variable denoting the day at which patient i experiences the first symptomatic blood-stage recurrence. For all patients $i \in G_{x,y}$, we assume all T_i to be independent and identically distributed with cumulative distribution function $I(t \mid \lambda_x, f_x, \varepsilon_y)$. Note that ε depends on the drug regimen y, and λ and f depend on the location x. If $j_i = 0$, then patient i experienced the first blood-stage recurrence at a time $T_i > t_i$ (data is right censored). The probability of this occurrence is

$$P(T_i > t_i) = 1 - I(t_i \mid \lambda_x, f_x, \varepsilon_y)\,.$$

Conversely, if $j_i = 1$, then patient i experienced the first blood-stage recurrence at a time $t_i - 1 < T_i \le t_i$ (data is interval censored). The probability of this occurrence is

$$P(t_i - 1 < T_i \le t_i) = I(t_i \mid \lambda_x, f_x, \varepsilon_y) - I(t_i - 1 \mid \lambda_x, f_x, \varepsilon_y)\,.$$

We define the log-likelihood of location x and trial arm y as

$$\begin{aligned} &\ell_{x,y}\left(\lambda_x, f_x, \varepsilon_y \mid \{t_i, j_i\}_{i\in G_{x,y}}\right) \\ &\quad = \sum_{i\in G_{x,y}} \ell\left(\lambda_x, f_x, \varepsilon_y \mid t_i, j_i\right) \\ &\quad = \sum_{\{i\in G_{x,y} \mid j_i = 0\}} \ln\left(P(T_i > t_i)\right) \\ &\qquad + \sum_{\{i\in G_{x,y} \mid j_i = 1\}} \ln\left(P(t_i - 1 < T_i \le t_i)\right) \\ &\quad = \sum_{\{i\in G_{x,y} \mid j_i = 0\}} \ln\left(1 - I(t_i \mid \lambda_x, f_x, \varepsilon_y)\right) \\ &\qquad + \sum_{\{i\in G_{x,y} \mid j_i = 1\}} \ln\left(I(t_i \mid \lambda_x, f_x, \varepsilon_y) - I(t_i - 1 \mid \lambda_x, f_x, \varepsilon_y)\right)\,. \end{aligned}$$

We fit distinct relapse and infection rates for each location, and distinct hypnozoiticidal efficacies for 7 mg/kg of PQ over 7 and 14 days to data from all locations and all trial arms simultaneously by summing the respective log-likelihoods

$$\ell(\text{PvRM} \mid \text{IMPROV data}) = \sum_x \sum_y \ell_{x,y}\left(\lambda_x, f_x, \varepsilon_y \mid \{t_i, j_i\}_{i \in G_{x,y}}\right). \quad (5)$$

The PvRM calibrated to the Commons et al meta-analysis[2] is shown in Figure 1, and the resulting posterior densities are shown in Figure S2.

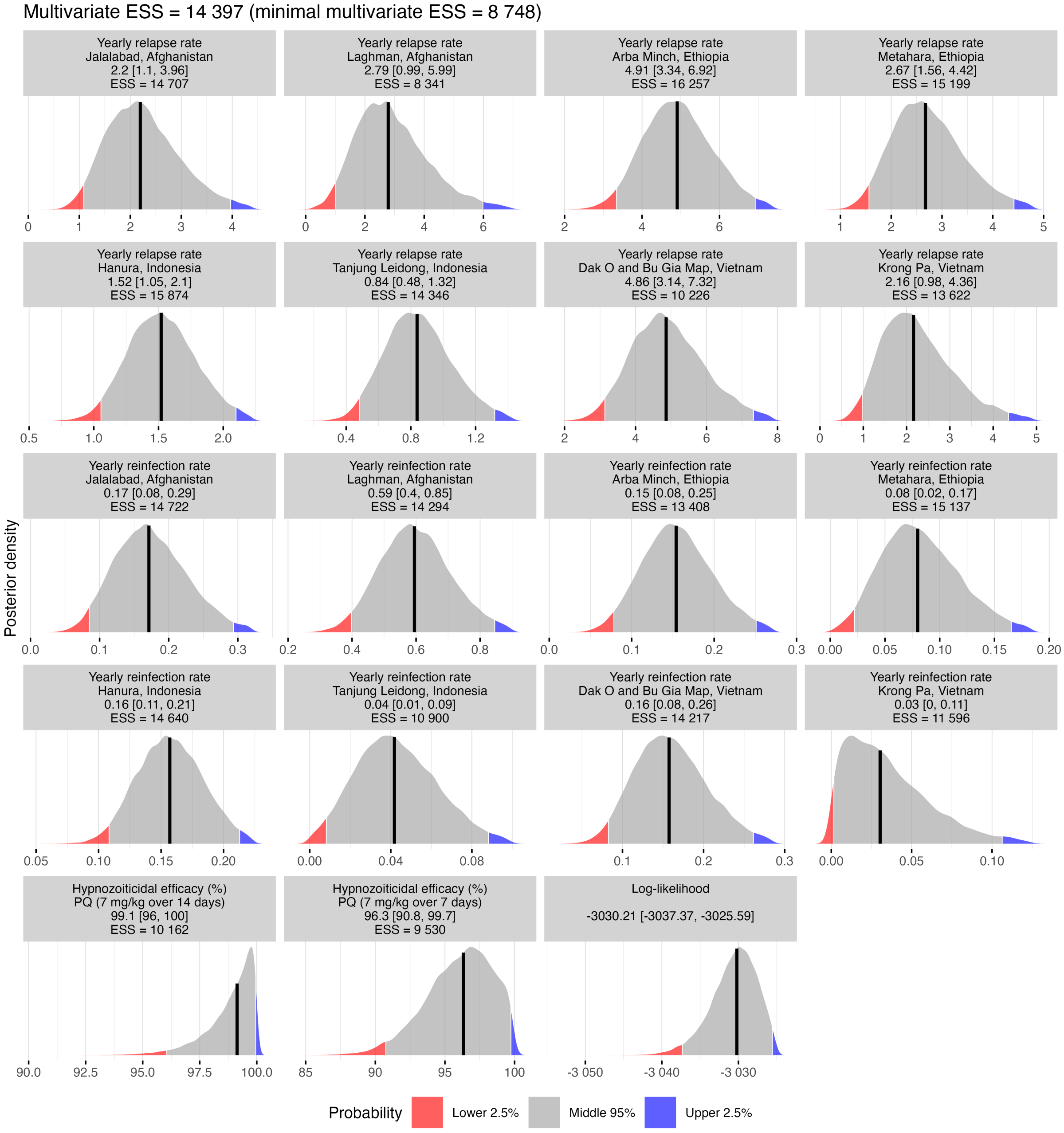

Figure S2: Posterior densities of all parameter estimates and the log-likelihood of the P. vivax Recurrence Model (PvRM) fit against patient-level data from the IMPROV clinical trial.[1] We adopted the all-or-nothing mechanism to model hypnozoite elimination. For each model, we ran six Markov Chain Monte Carlo (MCMC) chains with 50 000 iterations, thinning per 10 iterations and discarding 25% for burnin. Convergence of MCMC chains was assessed visually. (PQ) primaquine.

Location	8-AQ regimen	Day 42	Day 86	Day 176	Day 267	Day 356
Jalalabad, Afghanistan	No 8-AQs	44	21	12	8	8
Jalalabad, Afghanistan	PQ (7 mg/kg over 14 days)	91	55	39	31	28

Jalalabad, Afghanistan	PQ (7 mg/kg over 7 days)	83	52	36	33	27
Laghman, Afghanistan	No 8-AQs	20	10	8	5	3
Laghman, Afghanistan	PQ (7 mg/kg over 14 days)	42	36	30	24	17
Laghman, Afghanistan	PQ (7 mg/kg over 7 days)	39	36	27	22	11
Arba Minch, Ethiopia	No 8-AQs	50	26	15	10	4
Arba Minch, Ethiopia	PQ (7 mg/kg over 14 days)	128	120	76	53	21
Arba Minch, Ethiopia	PQ (7 mg/kg over 7 days)	122	102	71	50	18
Metahara, Ethiopia	No 8-AQs	34	22	11	8	3
Metahara, Ethiopia	PQ (7 mg/kg over 14 days)	84	81	51	40	22
Metahara, Ethiopia	PQ (7 mg/kg over 7 days)	81	77	50	35	17
Hanura, Indonesia	No 8-AQs	119	104	61	51	25
Hanura, Indonesia	PQ (7 mg/kg over 14 days)	222	210	191	175	79
Hanura, Indonesia	PQ (7 mg/kg over 7 days)	225	208	184	171	75
Tanjung Leidong, Indonesia	No 8-AQs	79	72	52	44	31
Tanjung Leidong, Indonesia	PQ (7 mg/kg over 14 days)	153	142	128	110	79
Tanjung Leidong, Indonesia	PQ (7 mg/kg over 7 days)	163	143	128	113	88
Dak O and Bu Gia Map, Vietnam	No 8-AQs	37	17	9	6	3
Dak O and Bu Gia Map, Vietnam	PQ (7 mg/kg over 14 days)	80	69	57	45	23
Dak O and Bu Gia Map, Vietnam	PQ (7 mg/kg over 7 days)	81	61	52	43	21
Krong Pa, Vietnam	No 8-AQs	21	12	7	5	4
Krong Pa, Vietnam	PQ (7 mg/kg over 14 days)	42	40	36	32	18
Krong Pa, Vietnam	PQ (7 mg/kg over 7 days)	42	42	40	34	18

Table S5: Numbers at risk for Kaplan-Meier plot Figure 1.

Log-likelihood function for the Commons et al meta-analysis

To estimate the hypnozoiticidal efficacy of 3·5 mg/kg of PQ, we used aggregated data from the Commons et al meta-analysis.[2] For each treatment group y, we know the number of enrolled patients n_y and the Kaplan-Meier estimate p_y for the risk of blood-stage recurrence by day $t_F = 180$. Then $k_y := round(p_y \cdot n_y)$ denotes the Kaplan-Meier estimate for the number of blood-stage recurrences by day t_F rounded to the nearest integer.

Assume each patient in treatment group y to have a probability $I(t_F \mid \lambda, f, \varepsilon_y)$ of having a blood-stage recurrence by day t_F. The probability of getting exactly k_y blood-stage recurrences by day t_F among n_y patients with the same recurrence probability $I(t_F \mid \lambda, f, \varepsilon_y)$ is binomially distributed. That is

$$k_y \sim \text{Binomial}\left(n_y,\, I(t_F \mid \lambda, f, \varepsilon_y)\right).$$

We define the log-likelihood of treatment group y as

$$\begin{aligned}
&\ell_y(\lambda, f, \varepsilon_y \mid k_y, n_y) \\
&\quad = \ln\left(P\left(k_y \mid n_y, I(t_F \mid \lambda, f, \varepsilon_y)\right)\right) \\
&\quad = \ln\left(\Gamma(n_y + 1)\right) - \ln\left(\Gamma(k_y + 1)\right) - \ln\left(\Gamma(n_y - k_y + 1)\right) \\
&\qquad + k_y \ln\left(I(t_F \mid \lambda, f, \varepsilon_y)\right) + (n_y - k_y)\ln\left(1 - I(t_F \mid \lambda, f, \varepsilon_y)\right).
\end{aligned}$$

The hypnozoiticidal efficacy of the 7 mg/kg PQ regimen was fixed to the median estimates from the previous fitting step A. We fit a relapse rate, an infection rate, and the hypnozoiticidal efficacy of 3·5 mg/kg of PQ to data from all trial groups simultaneously by summing the respective log-likelihoods

$$\ell(\text{PvRM} \,|\, \text{Commons et al data}) = \sum_{y} \ell_y\left(\lambda, f, \varepsilon_y \,|\, k_y, n_y\right). \qquad (6)$$

The PvRM calibrated to the Commons et al meta-analysis[2] is shown in Figure S3, and the resulting posterior densities are shown in Figure S4.

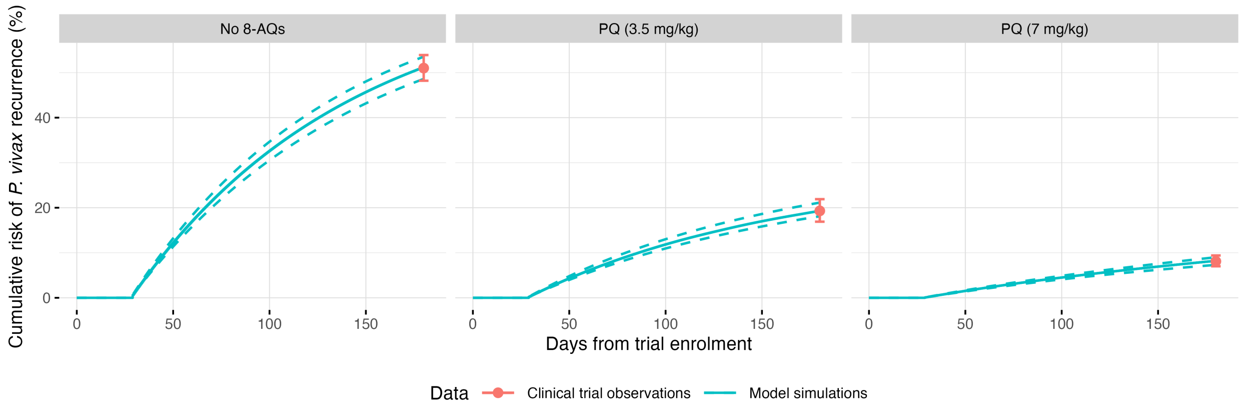

Figure S3: Calibration of the P. vivax Recurrence Model (PvRM) using the all-or-nothing mechanism to meta-analysis of patient-level clinical trial data.[2] The red points represent the Kaplan-Meier estimate for the risk of blood-stage recurrence by day 180, and the red bars denote the 95% confidence interval around the Kaplan-Meier estimates. The solid blue curves represent the posterior median model prediction, and the dashed blue curves the 95% credible intervals. All patients were treated with blood-stage drugs. (8-AQ) 8-aminoquinoline, (PQ) primaquine.

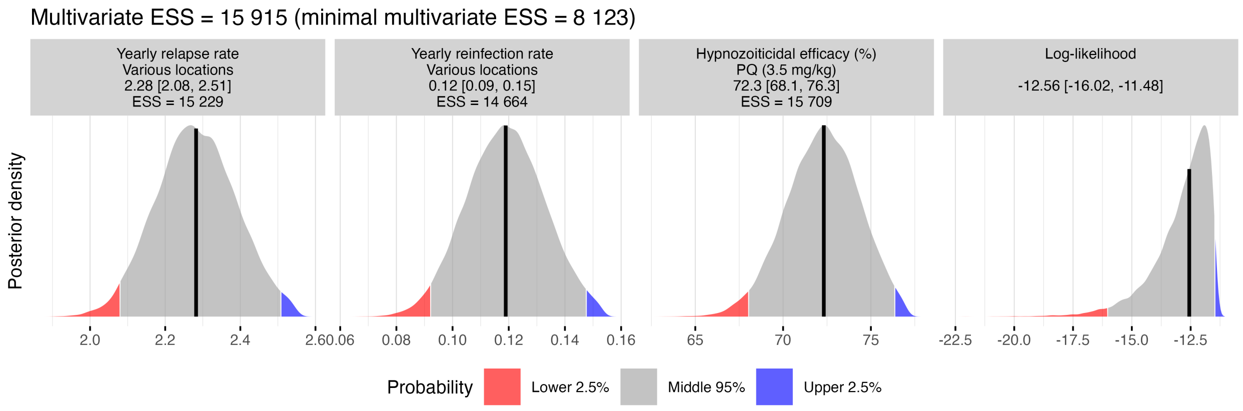

Figure S4: Posterior densities of all parameter estimates and the log-likelihood of the P. vivax Recurrence Model (PvRM) fit to meta-analysis of patient-level clinical trial data.[2] We adopted the all-or-nothing mechanism to model hypnozoite elimination. For each model, we ran six Markov Chain Monte Carlo (MCMC) chains with 50 000 iterations, thinning per 10 iterations and discarding 25% for burnin. Convergence of MCMC chains was assessed visually. (PQ) primaquine.

Log-likelihood function for the Watson et al meta-analysis

To estimate the hypnozoiticidal efficacy of 5 and 7·5 mg/kg of TQ, we used aggregated data from the Watson et al meta-analysis.[3] For each treatment group y, we know the number of enrolled patients n_y and the number k_y of patients with a blood-stage recurrence by day $t_F = 120$.

Assume each patient in treatment group y to have a probability $I\left(t_F \,|\, \lambda, f, \varepsilon_y\right)$ of having a blood-stage recurrence by day t_F. The probability of getting exactly k_y blood-stage recurrences by day t_F among n_y patients with the same recurrence probability $I\left(t_F \,|\, \lambda, f, \varepsilon_y\right)$ is binomially distributed. That is

$$k_y \sim \text{Binomial}\left(n_y,\, I\left(t_F \mid \lambda, f, \varepsilon_y\right)\right).$$

We define the log-likelihood of treatment group y as

$$\begin{aligned}
&\ell_y\left(\lambda, f, \varepsilon_y \mid k_y, n_y\right) \\
&\quad = \ln\left(P\left(k_y \mid n_y, I\left(t_F \mid \lambda, f, \varepsilon_y\right)\right)\right) \\
&\quad = \ln\left(\Gamma\left(n_y + 1\right)\right) - \ln\left(\Gamma\left(k_y + 1\right)\right) - \ln\left(\Gamma\left(n_y - k_y + 1\right)\right) \\
&\qquad + k_y \ln\left(I\left(t_F \mid \lambda, f, \varepsilon_y\right)\right) + \left(n_y - k_y\right)\ln\left(1 - I\left(t_F \mid \lambda, f, \varepsilon_y\right)\right).
\end{aligned}$$

The hypnozoiticidal efficacy of 3·5 mg/kg PQ was fixed to the median estimate from the previous fitting step. We fit a relapse rate, an infection rate, and the hypnozoiticidal efficacies of 5 and 7·5 mg/kg of TQ to data from all trial groups simultaneously by summing the respective log-likelihoods

$$\ell(\text{PvRM} \mid \text{Watson et al data}) = \sum_y \ell_y\left(\lambda, f, \varepsilon_y \mid k_y, n_y\right). \qquad (7)$$

The PvRM calibrated to the Watson et al meta-analysis[3] is shown in Figure S5, and the resulting posterior densities are shown in Figure S6.

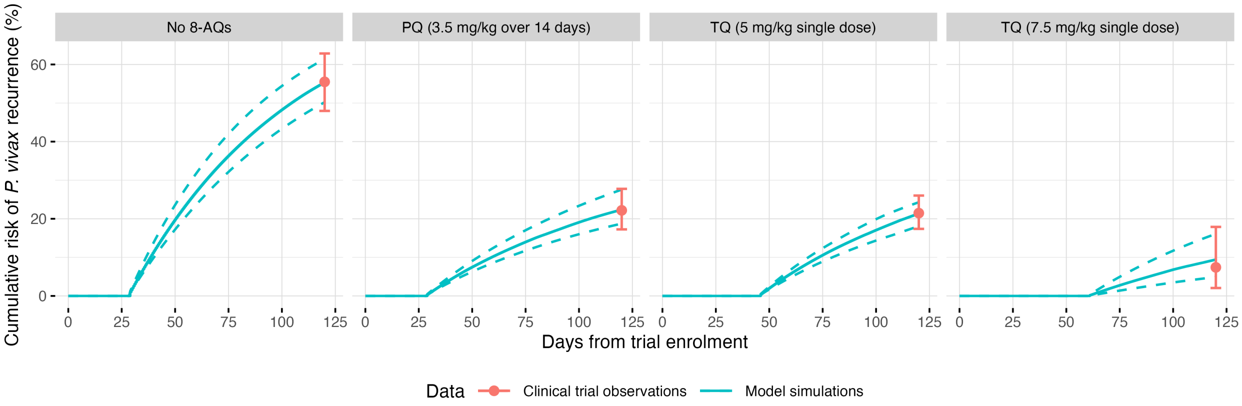

Figure S5: Calibration of the P. vivax Recurrence Model (PvRM) using the all-or-nothing mechanism to meta-analysis of patient-level clinical trial data.[3] The red points represent the proportion of patients with a blood-stage recurrence by day 120, and the red bars denote the 95% exact binomial confidence interval around this proportion. The solid blue curves represent the posterior median model prediction, and the dashed blue curves the 95% credible intervals. All patients were treated with blood-stage drugs. (8-AQ) 8-aminoquinoline, (PQ) primaquine, (TQ) tafenoquine.

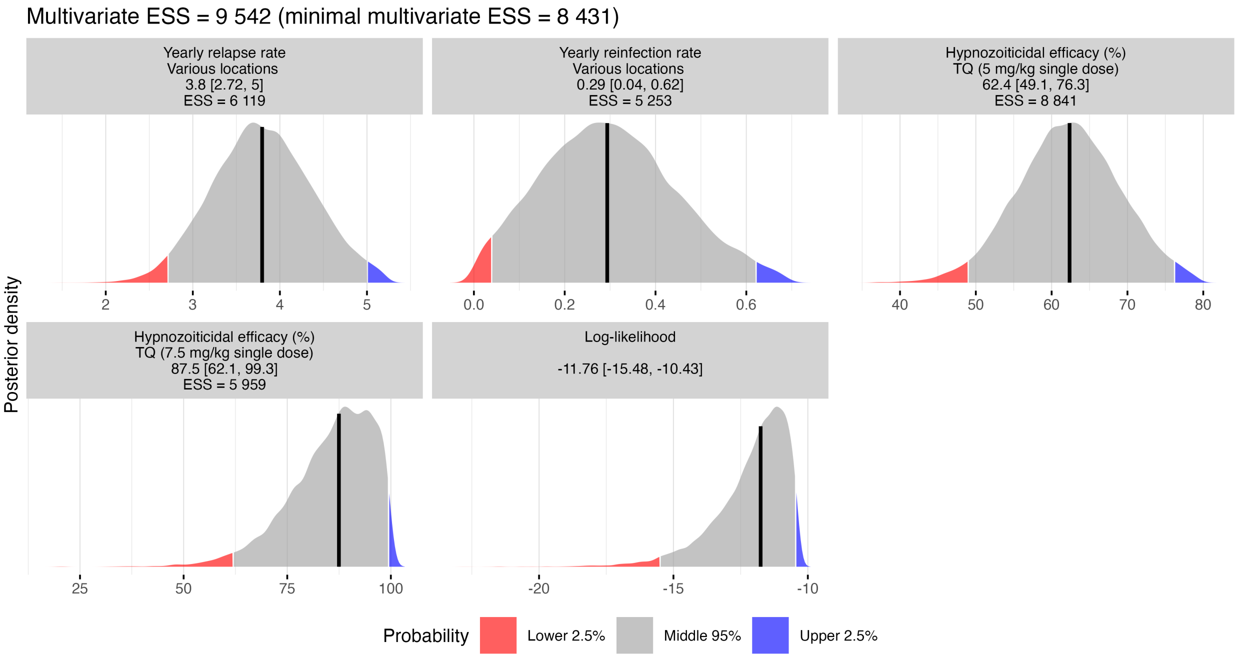

Figure S6: Posterior densities of all parameter estimates and the log-likelihood of the P. vivax Recurrence Model (PvRM) fit to meta-analysis of patient-level clinical trial data.[3] We adopted the all-or-nothing mechanism to model hypnozoite elimination. For each model, we ran six Markov Chain Monte Carlo (MCMC) chains with 50 000 iterations, thinning per 10 iterations and discarding 25% for burnin. Convergence of MCMC chains was assessed visually. (TQ) tafenoquine.

Additional results

Location	Yearly relapse rate	Yearly reinfection rate	Proportion of recurrences due to relapses (%)	Fitted to
Jalalabad, Afghanistan	2·2 [1·1, 3·96]	0·17 [0·08, 0·29]	92·8 [82·7, 97·2]	Patient-level trial data[1]
Laghman, Afghanistan	2·79 [0·99, 5·99]	0·59 [0·4, 0·85]	82·6 [59·5, 92·1]	Patient-level trial data[1]
Arba Minch, Ethiopia	4·91 [3·34, 6·92]	0·15 [0·08, 0·25]	97 [94·2, 98·5]	Patient-level trial data[1]
Metahara, Ethiopia	2·67 [1·56, 4·42]	0·08 [0·02, 0·17]	97·2 [92·7, 99·2]	Patient-level trial data[1]
Hanura, Indonesia	1·52 [1·05, 2·1]	0·16 [0·11, 0·21]	90·7 [84·8, 94·4]	Patient-level trial data[1]
Tanjung Leidong, Indonesia	0·84 [0·48, 1·32]	0·04 [0·01, 0·09]	95·3 [86·9, 99·2]	Patient-level trial data[1]
Dak O and Bu Gia Map, Vietnam	4·86 [3·14, 7·32]	0·16 [0·08, 0·26]	96·9 [94, 98·5]	Patient-level trial data[1]
Krong Pa, Vietnam	2·16 [0·98, 4·36]	0·03 [0, 0·11]	98·7 [93·9, 99·9]	Patient-level trial data[1]
Various locations	2·28 [2·08, 2·51]	0·12 [0·09, 0·15]	95 [93·6, 96·3]	Meta-analysis of patient-level trial data[2]
Various locations	3·8 [2·72, 5]	0·29 [0·04, 0·62]	92·8 [82·2, 99·1]	Meta-analysis of patient-level trial data[3]

Table S6: Median and 95% credible interval of parameter estimates of the P. vivax Recurrence Model (PvRM). We adopted the all-or-nothing mechanism to model hypnozoite elimination. For each model, we ran six Markov Chain Monte Carlo (MCMC) chains with 50 000 iterations, thinning per 10 iterations and discarding 25% for burnin. Convergence of MCMC chains was assessed visually.

Sensitivity analyses

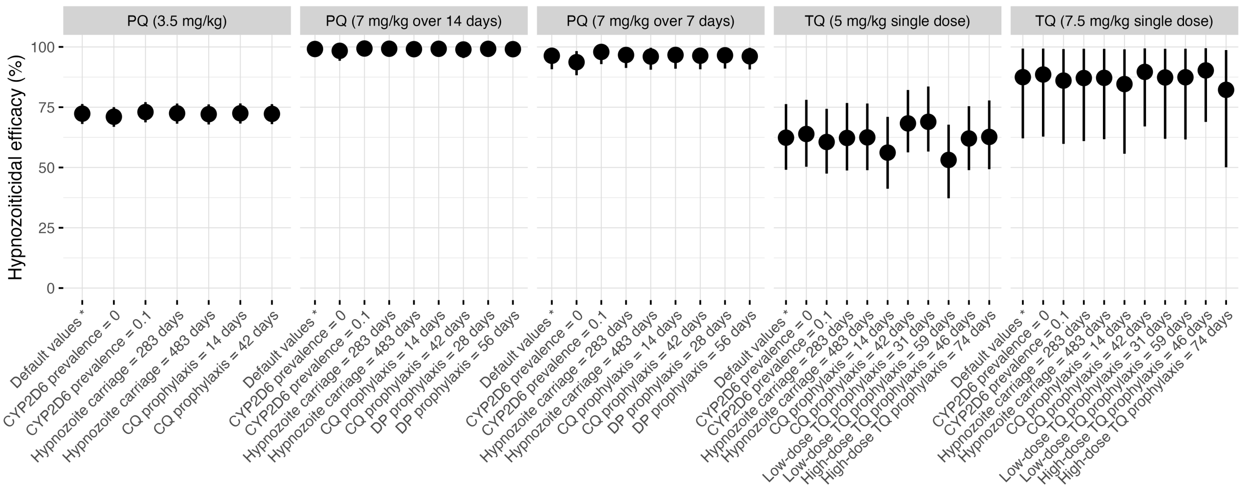

Figure S7: Hypnozoiticidal efficacy estimates obtained by varying key parameters of the P. vivax Recurrence Model (PvRM). Default and alternative values are listed in Table S4. Median and 95% credible interval across all 6 Markov Chain Monte Carlo (MCMC) chains. (CQ) chloroquine, (DP) dihydroartemisinin-piperaquine, (TQ) tafenoquine.

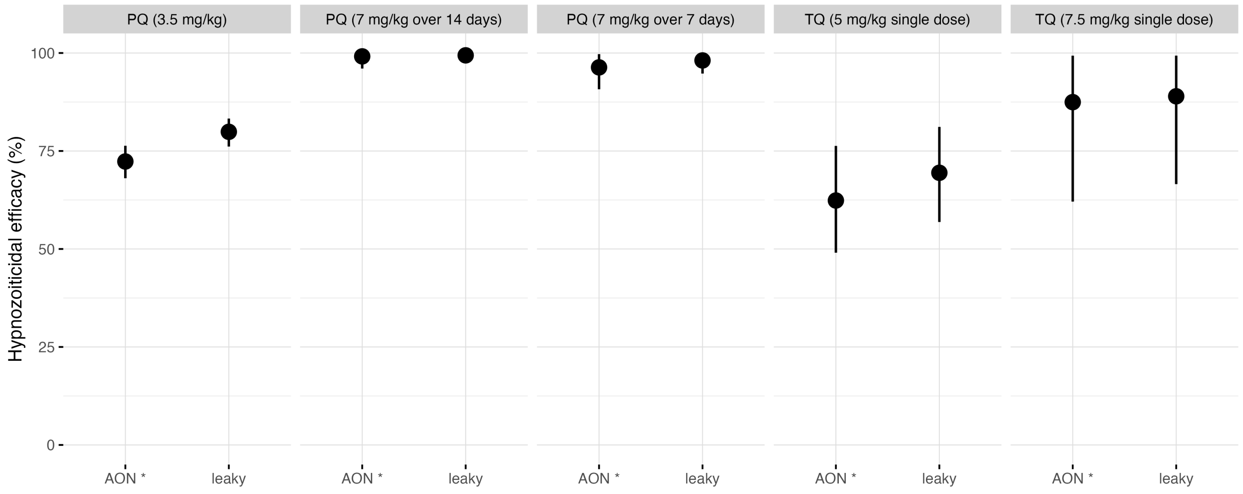

Figure S8: Hypnozoiticidal efficacy estimates under distinct assumptions on the mechanism of hypnozoite elimination of the P. vivax Recurrence Model (PvRM). (AON*) default model using the all-or-nothing mechanism, (leaky) alternative model using the leaky mechanism. Median and 95% credible interval across all 6 Markov Chain Monte Carlo (MCMC) chains.

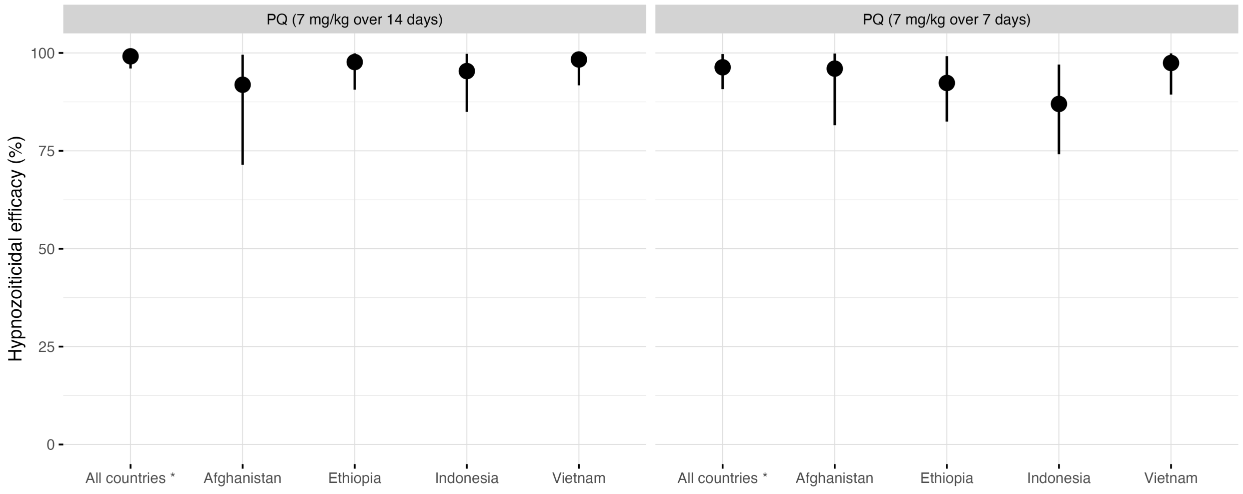

Figure S9: Hypnozoiticidal efficacy estimates obtained by fitting the P. vivax Recurrence Model (PvRM) separately to patient-level data from each country represented in the IMPROV clinical trial.[1] Median and 95% credible interval across all 6 Markov Chain Monte Carlo (MCMC) chains.

Extending the PvRM to operational conditions

This section essentially describes how to compute the cumulative incidence curves from Figure 2. The plots on the top row show cumulative incidence under trial conditions, i.e. assuming 100% eligibility and 100% adherence of participants in a hypothetical trial. The plots on the bottom row show cumulative incidence under operational conditions, where the assumptions on eligibility and adherence are relaxed. In particular, we assume that in routine clinical practice patients take the first 8-AQ dose under supervision and thus adherence to the single-dose drug TQ is perfect, while we assume 67 and 57% adherence to 7- and 14-day PQ regimens (Table S8).

Compute the proportion of a population eligible for each 8-aminoquinoline regimen

We want to compute what proportion of a population is eligible for each of the investigated 8-AQ regimens under operational conditions (Table 1). We assume a population sharing the same characteristics as the PvIBM's synthetic population (Table S8, Table S10). Age follows a truncated exponential distribution with mean age 22·5 years and maximum age 80 years. Since mortality is not age-specific, the proportion of a population aged less than a years is

$$P(\text{age} < a) = \frac{1 - e^{-\frac{a}{22\cdot5}}}{1 - e^{-\frac{80}{22\cdot5}}}.$$

Females between the ages of 16 and 45 are considered fertile, and 12·5% of them are assumed to be pregnant or breastfeeding a baby of <6 months at any given time. Thus, the proportion of females who are pregnant or breastfeeding is given by

$$P(\text{pregnant or breastfeeding} \mid \text{female}) := 0 \cdot 125\big(P(\text{age} < 45) - P(\text{age} < 16)\big).$$

Let q be the proportion of males with a deficient genotype. Since the gene for G6PD deficiency is x-linked, the remaining proportion $1 - q$ of males will have a normal genotype. According to the Hardy-Weinberg principle, a proportion q^2 of females will be homozygous deficient, a proportion $2q(1-q)$ heterozygous deficient, and the remaining proportion $(1-q)^2$ homozygous normal. Following Nekkab et al[13], we assume the G6PD activity of deficient, heterozygous deficient and normal subgroups to follow a Gaussian distribution with distinct mean μ and standard deviation σ. Let $F(\cdot \mid \mu, \sigma)$ be the cumulative distribution function of the normal distribution with mean μ and standard deviation σ. The proportion of males with a G6PD activity below a certain threshold θ is

$$P(\text{G6PD activity} < \theta \mid \text{male}) = q \cdot F\big(\theta \mid \mu_{def}, \sigma_{def}\big) + (1-q) \cdot F(\theta \mid \mu_{nor}, \sigma_{nor}).$$

The proportion of females with a G6PD activity below a certain threshold θ is

$$P(\text{G6PD activity} < \theta \mid \text{female}) \\ = q^2 \cdot F\big(\theta \mid \mu_{def}, \sigma_{def}\big) + 2\,q\,(1-q) \cdot F(\theta \mid \mu_{het}, \sigma_{het}) + (1-q)^2 \cdot F(\theta \mid \mu_{nor}, \sigma_{nor}).$$

Assuming an equal sex ratio, the proportion of the population that is not pregnant, not breastfeeding, aged $> a$ and with a G6PD activity $> \theta$ is

$$\frac{1}{2}\big(1 - P(\text{age} < a) - P(\text{pregnant or breastfeeding} \mid \text{female})\big)\big(1 - P(\text{G6PD activity} < \theta \mid \text{female})\big) \\ + \frac{1}{2}\big(1 - P(\text{age} < a)\big)\big(1 - P(\text{G6PD activity} < \theta \mid \text{male})\big).$$

8-AQ regimen	Eligible population (%)
No 8-AQ	100·0
PQ given at a daily dose of 0·25 or 0·5 mg/kg	92·0
PQ given at a daily dose of 1 mg/kg	80·6
TQ	75·1

Table S7: Proportion of a population that is eligible for various 8-AQ regimens. The eligibility criteria for different 8-AQ regimens are listed in Table 1. We assume a population sharing the same characteristics as the PvIBM's synthetic population (Table S8, Table S10). (8-AQ) 8-aminoquinoline, (PQ) primaquine, (TQ) tafenoquine.

Computing the cumulative incidence of blood-stage recurrence under operational conditions

Let m denote the mechanism of hypnozoite elimination, c the proportion of the population who are low CYP2D6 metabolisers, γ the rate of hypnozoite clearance, λ the infection rate, and f the relapse rate. Let y be an 8-AQ regimen, and denote u_y the proportion of the population who are eligible for y (computed in the preceeding paragraph), v_y the proportion of the population who fully adhere to y (values listed in Table S8), d_y the duration of post-treatment prophylaxis of y, and ε_y the hypnozoiticidal efficacy of y.

Let $I_{OC}(t\,|\,y) = I_{OC}(t\,|\,m, c, \gamma, d_y, \lambda, f, \varepsilon_y, u_y, v_y)$ denote the cumulative incidence following treatment of symptomatic *P. vivax* with 8-AQ regimen y under operational conditions.

If y denotes a schizontocidal drug regimen without use of 8-AQs, then $u_y = 1$, $\varepsilon_y = 0$ and $v_y = 1$ and

$$I_{OC}(t\,|\,y = \text{No 8-AQ}) = u_y \cdot I(t\,|\,m, c, \gamma, d_y, \lambda, f, \varepsilon_y \cdot v_y) = I(t\,|\,m, c, \gamma, d_y, \lambda, f, 0)\,.$$

If y denotes the magic bullet drug regimen, then $u_y = 1$, $\varepsilon_y = 1$ and $v_y = 1$ and

$$I_{OC}(t\,|\,y = \text{Magic bullet}) = u_y \cdot I(t\,|\,m, c, \gamma, d_y, \lambda, f, \varepsilon_y \cdot v_y) = I(t\,|\,m, c, \gamma, d_y, \lambda, f, 1)\,.$$

If y denotes a drug regimen where PQ is given at a daily dose of 0·25 or 0·5 mg/kg, then a schizontocidal drug z is given to patients who are not eligible for drug regimen y

$$\begin{aligned} &I_{OC}(t\,|\,y = \text{PQ given at} \leq 0 \cdot 5 \text{ mg/kg}) \\ &\quad = u_y \cdot I(t\,|\,m, c, \gamma, d_y, \lambda, f, \varepsilon_y \cdot v_y) \;+\; (1 - u_y) \cdot I_{OC}(t\,|\,z = \text{No 8-AQ})\,. \end{aligned}$$

If y denotes a drug regimen including TQ or where PQ is given at a daily dose of 1 mg/kg, then an alternative drug regimen z consisting of a schizontocidal drug plus PQ at 3·5 mg/kg over 7 days is given to patients who are not eligible for drug regimen y

$$\begin{aligned} &I_{OC}(t\,|\,y = \text{TQ or PQ given at 1 mg/kg}) \\ &\quad = u_y \cdot I(t\,|\,m, c, \gamma, d_y, \lambda, f, \varepsilon_y \cdot v_y) \\ &\qquad + (1 - u_y) \cdot I_{OC}(t\,|\,z = \text{PQ given at } 3 \cdot 5 \text{ mg/kg over 7 days})\,. \end{aligned}$$

Quantifying the community-level impact of radical cure case management using the *P. vivax* Individual-Based Model (PvIBM)

Model overview

To simulate *P. vivax* transmission at the community level, we used a stochastic individual-based model, called here PvIBM, developed by White et al[14] and Nekkab et al.[13] The frequency with which an individual is infected depends on mosquito density, an individual's heterogeneity in exposure to mosquito bites, and age-dependent biting differences (individuals with a smaller skin surface get bitten less frequently). An individual who is inoculated with *P. vivax* through an infectious mosquito bite will develop a primary blood-stage infection and acquire a brood of hypnozoites within the liver. Individuals can accumulate multiple hypnozoite broods from distinct inoculation events. Each hypnozoite brood can cause relapses at a rate f or be cleared naturally at a rate γ.

Individuals acquire immunity with each new blood-stage infection. Two types of immunity are considered. An individual's level of anti-parasite immunity determines the probability with which a new blood-stage infection will be detectable by light-microscopy (as opposed to only being detectable by PCR) and controls the rate of clearance of blood-stage infections. The level of clinical immunity, on the other hand, determines the probability with which a new light-microscopy detectable infection will be symptomatic (a clinical episode is characterised by a fever ≥ 38°C in the preceding 48 hours and a parasite density ≥ 500/ml).

Individuals with clinical malaria seek treatment according to a certain probability (which we will call case management coverage later). Schizontocidal drugs eliminate all blood-stage parasites and subsequently protect the individual from further blood-stage infections for a certain amount of time (post-treatment prophylaxis of the schizontocidal drug). If given, 8-AQs eliminate all hypnozoite broods with a certain probability depending on the 8-AQ regimen, whether the individual is a low CYP2D6 metaboliser, and whether the individual adhered to the full 8-AQ course. During the post-treatment prophylaxis of the 8-AQ regimen, hypnozoites can not reactivate and no new hypnozoite broods can be established in the individual's liver.

The population dynamics of *Anopheles* mosquitoes are described via a compartmental model. The larval stage is constrained by density-dependent competition in larval breeding sites whose size varied seasonally. Adult female mosquitoes follow feeding, resting and oviposition cycles informed by entomological observations on their life expectancy, the proportion of blood meals taken on humans (human blood index), their preferred biting time (day time vs. night time biting), and their indoor resting preferences.

The PvIBM is calibrated to epidemiological[14] and entomological surveys[15] from Papua New Guinea, the Solomon Islands, and Nigeria.

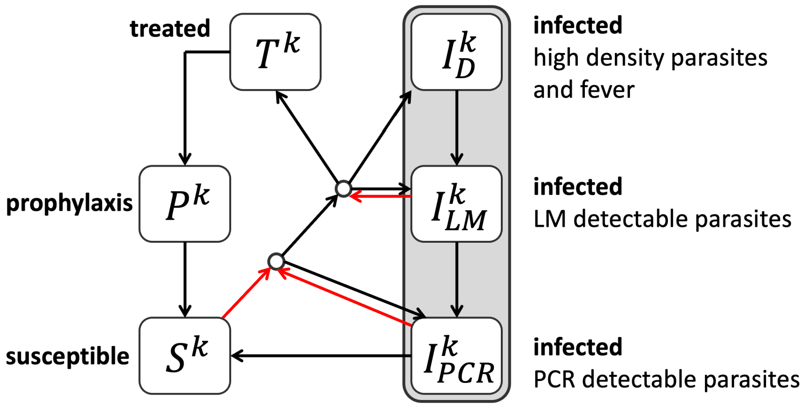

Figure S10: Compartmental representation of the P. vivax Individual-Based Model (PvIBM) developed by White et al[14] and Nekkab et al.[13] Infected individuals can be in one of three compartments depending on whether blood-stage parasitaemia is detectable by PCR (I_{PCR}), light microscopy (I_{LM}) or has high density with accompanying fever (I_D). A proportion of individuals that progress to a symptomatic episode of P. vivax will undergo treatment with a blood-stage drug (T) leading to clearance of blood-stage parasitaemia and a period of prophylactic protection (P) before returning to the susceptible state (S). The superscript k denotes the number of broods of relapse-causing hypnozoites in the liver. Figure adapted from White et al.[14]

Model parameters

Description	Value	References
Mean population age (in days)	8210	Assumption
Maximum population age (in days)	29200	Assumption
Proportion of females in the population	0·5	Assumption
Proportion of adult women that are pregnant or breastfeeding a baby of <6 months	0·125	Assumption
Minimum age for start of pregnancy (in days)	5840	Assumption
Maximum age for start of pregnancy (in days)	16400	Assumption
Duration of pregnancy and breastfeeding (in days)	450	Assumption
Age-dependent biting parameter	2920	[16,17]
Age-dependent biting parameter	0·85	[16,17]
Probability of mosquito-to-human transmission	0·5	Assumption
Probability of (PCR-detectable) human-to-mosquito transmission	0·035	[18]
Probability of (LM-detectable) human-to-mosquito transmission	0·1	[18]
Probability of human-to-mosquito transmission	0·8	[18]
Probability of human-to-mosquito transmission	0·4	[18]
Duration of latency in the liver (in days)	10	[19]
Rate of recovery from symptomatic disease	0·2	Assumption[14]
Rate of progression through treatment	1	[20]
Duration of PCR-detectable infection (full immunity)	10	Assumption[14]
Rate of decay of anti-parasite immunity	0·000274	Assumption[14]
Rate of decay of clinical immunity	0·0000913	Assumption[14]
Mean duration of hypnozoite carriage (in days)	383	[9]
Mean G6PD activity - homozygous normal females and hemizygous normal males	10·2	[13]
Standard deviation in G6PD activity - homozygous normal females and hemizygous normal males	2·39	[13]
Mean G6PD activity - heterozygous deficient females	4·6	[13]
Standard deviation in G6PD activity - heterozygous deficient females	1·67	[13]
Mean G6PD activity - homozygous deficient females and hemizygous deficient males	0·48	[13]
Standard deviation in G6PD activity - homozygous deficient females and hemizygous deficient males	0·31	[13]
SD Biosensor STANDARD G6PD test - sensitivity in those with < 30% G6PD activity	100	[21]
SD Biosensor STANDARD G6PD test - specificity in those with < 30% G6PD activity	97	[21]
SD Biosensor STANDARD G6PD test - sensitivity in those with < 70% G6PD activity	95·5	[21]
SD Biosensor STANDARD G6PD test - specificity in those with < 70% G6PD activity	97	[21]
CQ efficacy	1	Assumption
CQ efficacy when given with PQ	1	Assumption
Duration of CQ prophylaxis (in days)	28	[10]
Duration of DP prophylaxis (in days)	42	[10]
Magic bullet - hypnozoiticidal efficacy	1	Assumption
Magic bullet - prophylaxis (in days)	28	Assumption
Magic bullet - probability of full adherence	1	Assumption
Magic bullet - lower age boundary for treatment prescription	0	Assumption
Magic bullet - presence of risk for G6PD deficient patients	0	Assumption
Magic bullet - ineffective for CYP2D6 low-metaboliser patients	0	Assumption
Magic bullet - presence of risk for pregnant and breastfeeding women	0	Assumption
Magic bullet - test for G6PD deficiency before administering treatment	0	Assumption
PQ over 7 days - duration of prophylaxis (in days)	8	Assumption
PQ over 14 days - duration of prophylaxis (in days)	15	Assumption
PQ over 7 days - adherence to full treatment course	0·67	[22]
PQ over 14 days - adherence to full treatment course	0·57	Assumption
PQ - lower age boundary for treatment prescription (in days)	180	[23]
PQ - presence of risk for G6PD deficient patients	1	[23]

PQ - ineffective for CYP2D6 low-metaboliser patients	1	[5–7]
PQ - presence of risk for pregnant and breastfeeding women (baby's G6PD status unknown)	1	[23]
PQ - test for G6PD deficiency before administering treatment	1	[24]
Low-dose TQ - duration of prophylaxis (in days)	45	[11]
High-dose TQ - duration of prophylaxis (in days)	60	Assumption
TQ - adherence to full treatment course	1	Assumption
TQ - lower age boundary for treatment prescription (in days)	730	Assumption
TQ - presence of risk for G6PD deficient patients	1	[25]
TQ - ineffective for CYP2D6 low-metaboliser patients	0	[6]
TQ - presence of risk for pregnant and breastfeeding women (baby's G6PD status unknown)	1	[25]
TQ - test for G6PD deficiency before administering treatment	1	[24]
Mosquito death rate (daily)	0·167	[13]
Duration of sporogony (in days)	8	[13]
Duration of early larval instar stage (in days)	6·64	[15]
Duration of late larval instar stage (in days)	3·72	[15]
Duration of pupae stage (in days)	0·64	[15]
Mortality rate of early larval instars (when density is low)	0·034	[15]
Mortality rate of late larval instars (when density is low)	0·035	[15]
Mortality rate of pupae	0·25	[15]
Number of eggs laid per day per mosquito	21·2	[15]
Effect of density dependence on late instars w.r.t. early instars	13·2	[15]
Human blood index (proportion of blood meals taken on humans)	0·5	[13]
Proportion of endophilic mosquitoes (resting indoors after feeding)	0·9	[13]
Proportion of bites taken on humans indoors	0·2	[13]
Proportion of bites taken on humans in bed	0·2	[13]
Time spent foraging for a blood meal	0·68	[13]
Time spent digesting blood meal (duration of gonotrophic cycle)	3	[26]

Table S8: Summary of fixed parameters for the P. vivax Individual-Based Model (PvIBM). (PCR) polymerase chain reaction, (LM) light microscopy, (G6PD) glucose-6-phosphate dehydrogenase, (CYP2D6) cytochrome P450 2D6, (CQ) chloroquine, (DP) dihydroartemisinin-piperaquine, (PQ) primaquine, (TQ) tafenoquine.

Description	Median and 95% CI
Heterogeneity in exposure, standard deviation on a log scale	1·536 [1·395, 1·668]
Duration of LM-detectable infection	16·02 [13·99, 18·21]
Duration of PCR-detectable infection (no immunity)	52·72 [33·47, 84·94]
Anti-parasite immunity - scale parameter	9·856 [6·469, 15·784]
Anti-parasite immunity - shape parameter	3·871 [2·592, 6·319]
Acquisition of anti-parasite immunity - scale parameter	42·1 [35·94, 49·57]
Probability of LM-detectable infection (no immunity)	0·927 [0·827, 0·99]
Probability of LM-detectable infection (full immunity)	0·011 [0·006, 0·016]
Anti-parasite immunity - scale parameter	18·78 [16·6, 21·61]
Anti-parasite immunity - shape parameter	3·368 [2·936, 3·933]
Acquisition of clinical immunity - scale parameter	4·322 [2·99, 6·227]
Probability of clinical disease (no immunity)	0·958 [0·861, 0·995]
Probability of clinical disease (full immunity)	0·007 [0·002, 0·016]
Acquisition of clinical immunity - scale parameter	24·51 [20·86, 29·31]
Acquisition of clinical immunity - shape parameter	5·71 [3·747, 8·39]
New-born immunity relative to mother's	0·303 [0·111, 0·661]
Duration of maternal immunity (in days)	48·96 [23·13, 121·54]

Table S9: Summary of posterior parameter draws obtained from the calibration of the P. vivax Individual-Based Model (PvIBM) by White et al.[14] (PCR) polymerase chain reaction, (LM) light microscopy.

Description	Default value	Alternative values
Yearly EIR at equilibrium	1	0·1, 10
Relapse periodicity (in days)	41	69, 120
Transmission level during dry season as compared to yearly average	0·1	0·05, 1
Proportion of the year that is dry season	0·5	0·25, 0·6
Proportion of symptomatic cases treated	0·9	0·5, 0·7, 1
Prevalence of hemizygous G6PD deficient males (genotype)	0·05	0, 0·1
Prevalence of CYP2D6 low metabolisers	0·05	0, 0·1
In case of a relapse - Factor for probability of LM-detectable infection	1	0·25, 0·5, 0·75
In case of a relapse - Factor for probability of symptomatic disease	1	0·25, 0·5, 0·75
In case of a relapse - Factor for increment of anti-parasite immunity	1	0·25, 0·5, 0·75
In case of a relapse - Factor for increment of clinical immunity	1	0·25, 0·5, 0·75
PQ over 7 days - adherence to full treatment course	0·67	0·2, 0·9
PQ over 14 days - adherence to full treatment course	0·57	0·1, 0·8

Table S10: Default and alternative parameter values for the P. vivax Individual-Based Model (PvIBM). (PQ) primaquine.

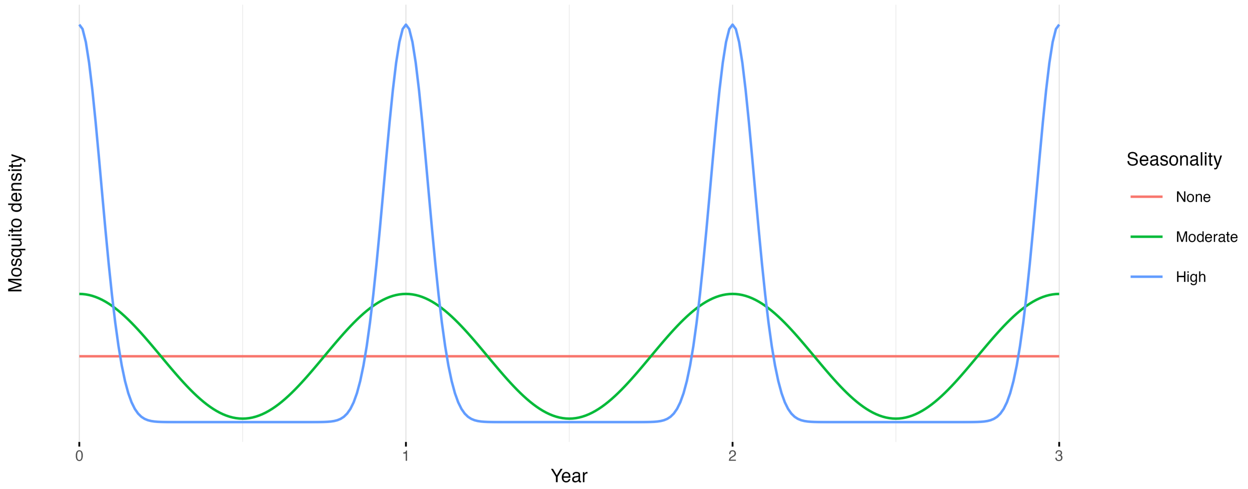

Figure S11: Seasonality patterns in mosquito density implemented in the P. vivax Individual-Based Model (PvIBM).

Additional results

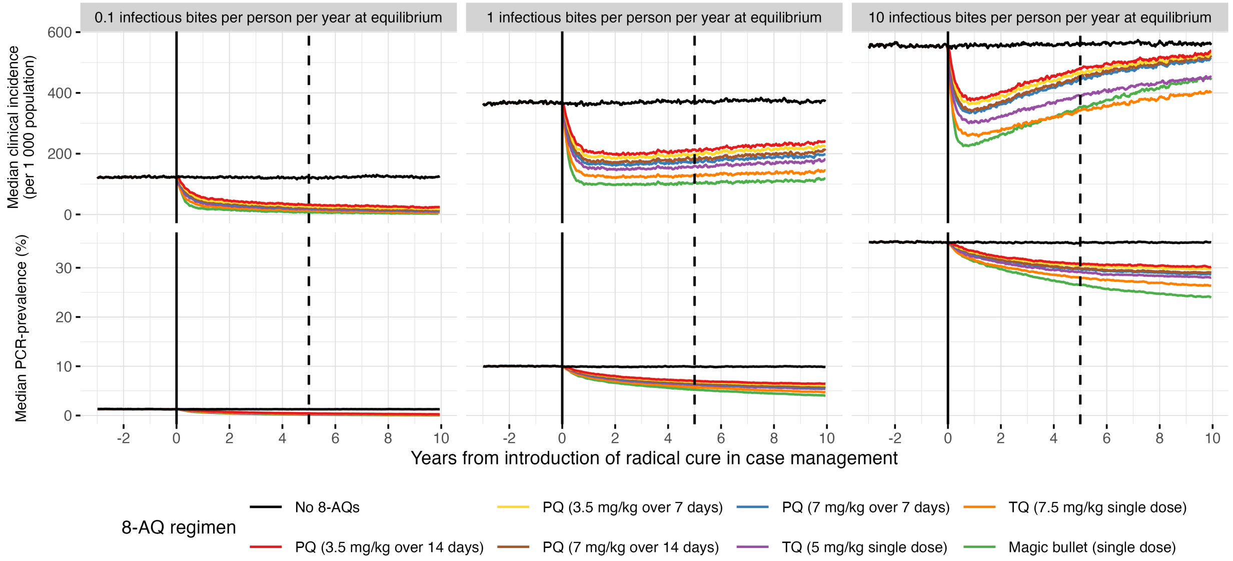

Figure S12: Simulated P. vivax transmission in a community with no malaria seasonality. Clinical incidence is shown in the top row, PCR-prevalence in the bottom row. The columns denote different transmission intensities. Radical cure is introduced in year 0 (solid vertical lines) and its impact is evaluated in year 5 (dashed vertical lines). Clinical incidence and PCR-prevalence are the medians of 100 independent simulations per scenario using the P. vivax Individual-Based Model (PvIBM). All patients were assumed to be treated with chloroquine (CQ) for blood-stage activity. (8-AQ) 8-aminoquinoline, (PQ) primaquine, (TQ) tafenoquine.

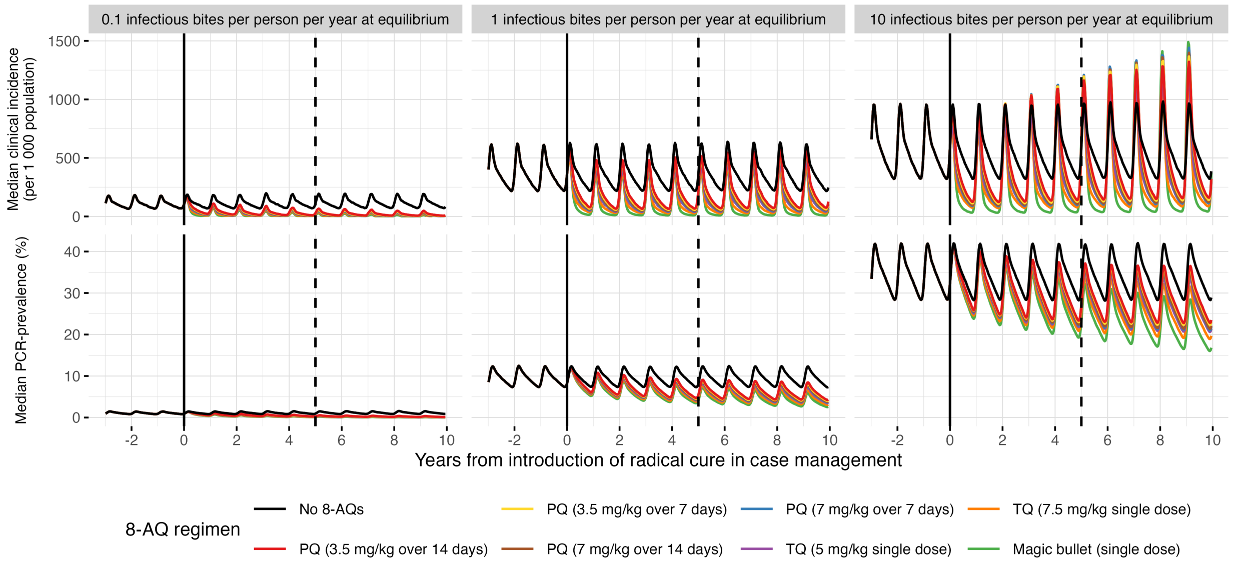

Figure S13: Simulated P. vivax transmission in a community with high malaria seasonality. Clinical incidence is shown in the top row, PCR-prevalence in the bottom row. The columns denote different transmission intensities. Radical cure is introduced in year 0 (solid vertical lines) and its impact is evaluated in year 5 (dashed vertical lines). Clinical incidence and PCR-prevalence are the medians of 100 independent simulations per scenario using the P. vivax Individual-Based Model (PvIBM). All patients were assumed to be treated with chloroquine (CQ) for blood-stage activity. (8-AQ) 8-aminoquinoline, (PQ) primaquine, (TQ) tafenoquine.

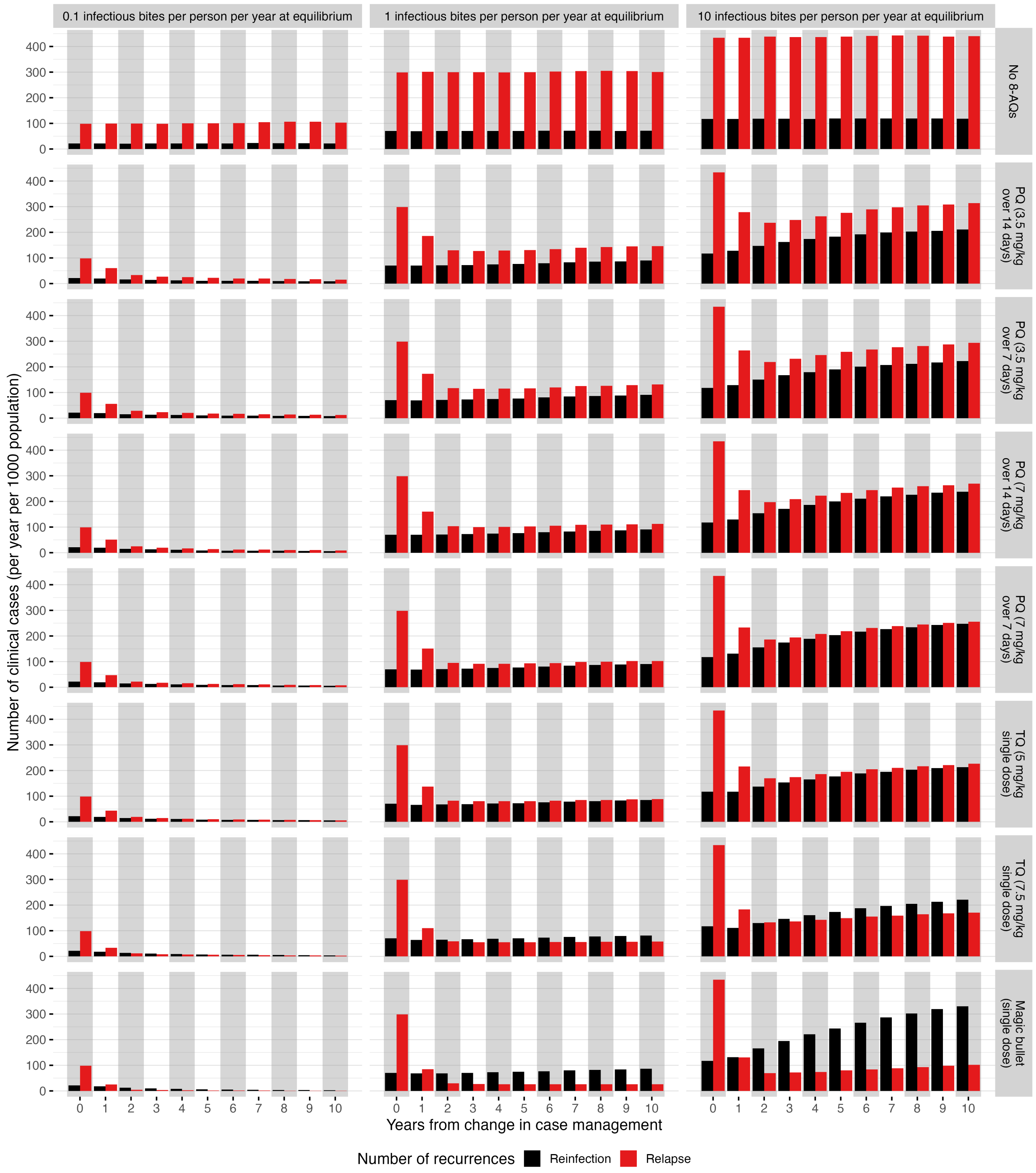

Figure S14: Yearly number of clinical relapses and reinfections. The columns denote different transmission intensities, rows different radical cure regimens. Bars depict medians of 100 independent simulations per scenario using the P. vivax Individual-Based Model (PvIBM). All patients were assumed to be treated with chloroquine (CQ) for blood-stage activity. (8-AQ) 8-aminoquinoline, (PQ) primaquine, (TQ) tafenoquine.

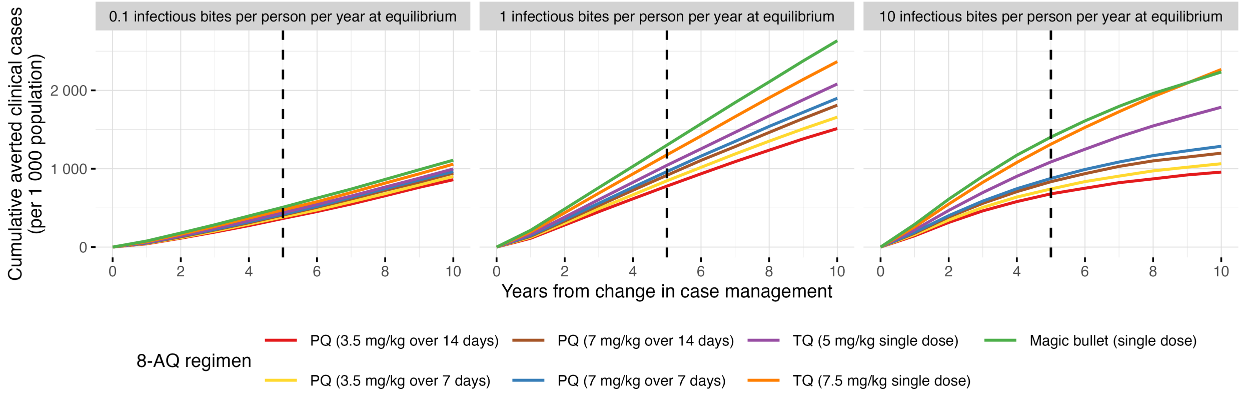

Figure S15: Cumulative averted clinical cases of P. vivax malaria. The columns denote different transmission intensities. Radical cure is introduced in year 0 and its impact is evaluated in year 5 (dashed vertical lines). Curves depict medians of 100 independent simulations per scenario using the P. vivax Individual-Based Model (PvIBM). All patients were assumed to be treated with chloroquine (CQ) for blood-stage activity. (8-AQ) 8-aminoquinoline, (PQ) primaquine, (TQ) tafenoquine.

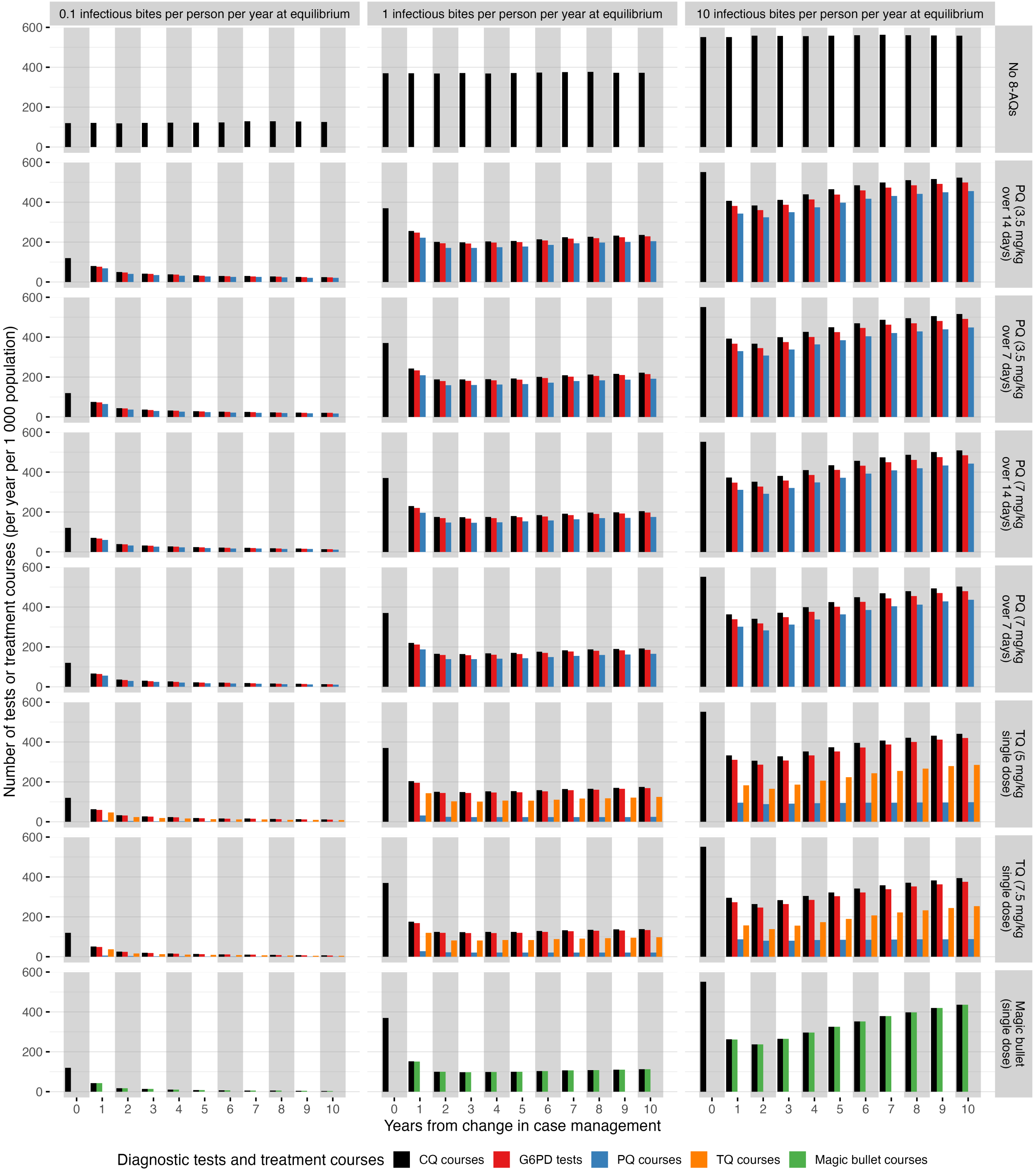

Figure S16: Yearly number of tests or treatment courses for P. vivax case management. The columns denote different transmission intensities, rows different radical cure regimens. Bars depict medians of 100 independent simulations per scenario using the P. vivax Individual-Based Model (PvIBM). All patients were assumed to be treated with chloroquine (CQ) for blood-stage activity. (8-AQ) 8-aminoquinoline, (PQ) primaquine, (TQ) tafenoquine.

Sensitivity analyses

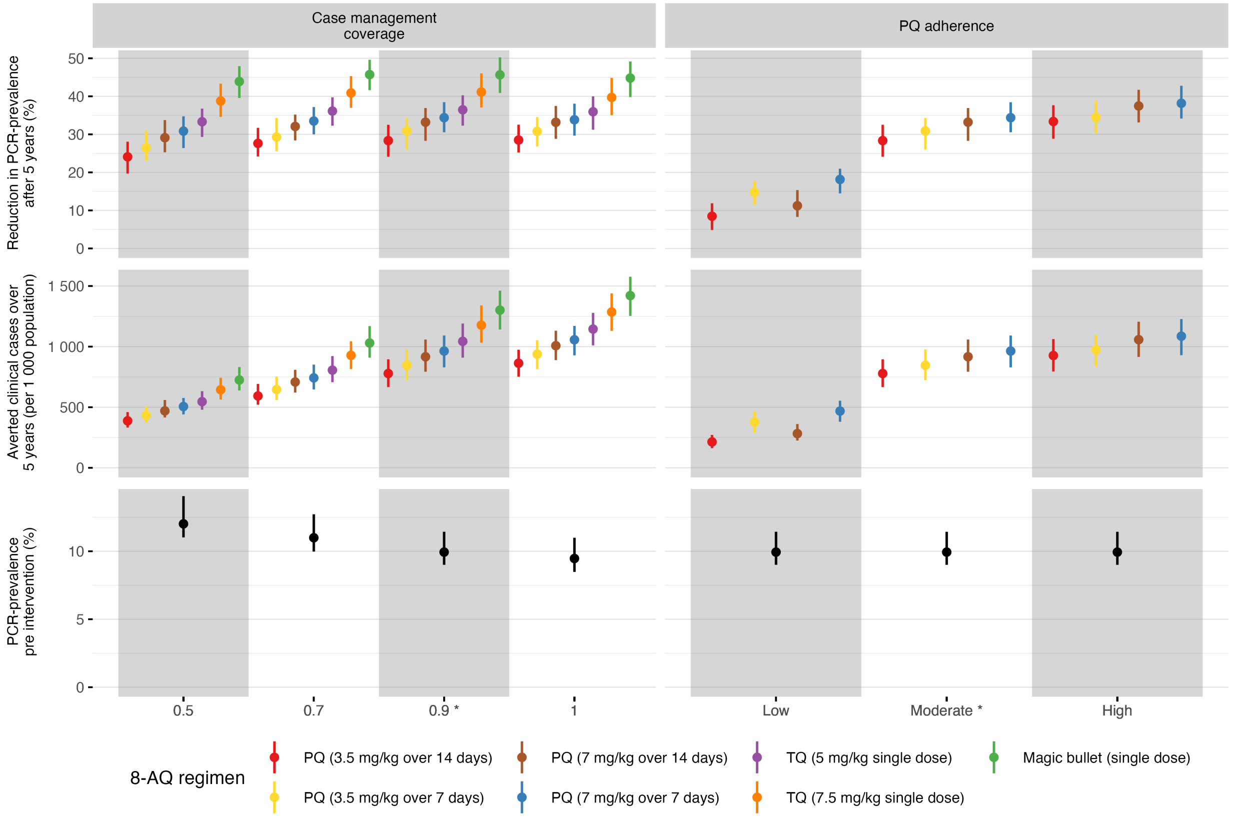

Figure S17: Estimates of radical cure impact obtained by varying key intervention characteristics. Default values (denoted with an asterisk *) and alternative values are listed in Table S10. Medians and 95% confidence intervals of 100 independent simulations per scenario using the P. vivax Individual-Based Model (PvIBM). All patients were assumed to be treated with chloroquine (CQ) for blood-stage activity. (8-AQ) 8-aminoquinoline, (PQ) primaquine, (TQ) tafenoquine.

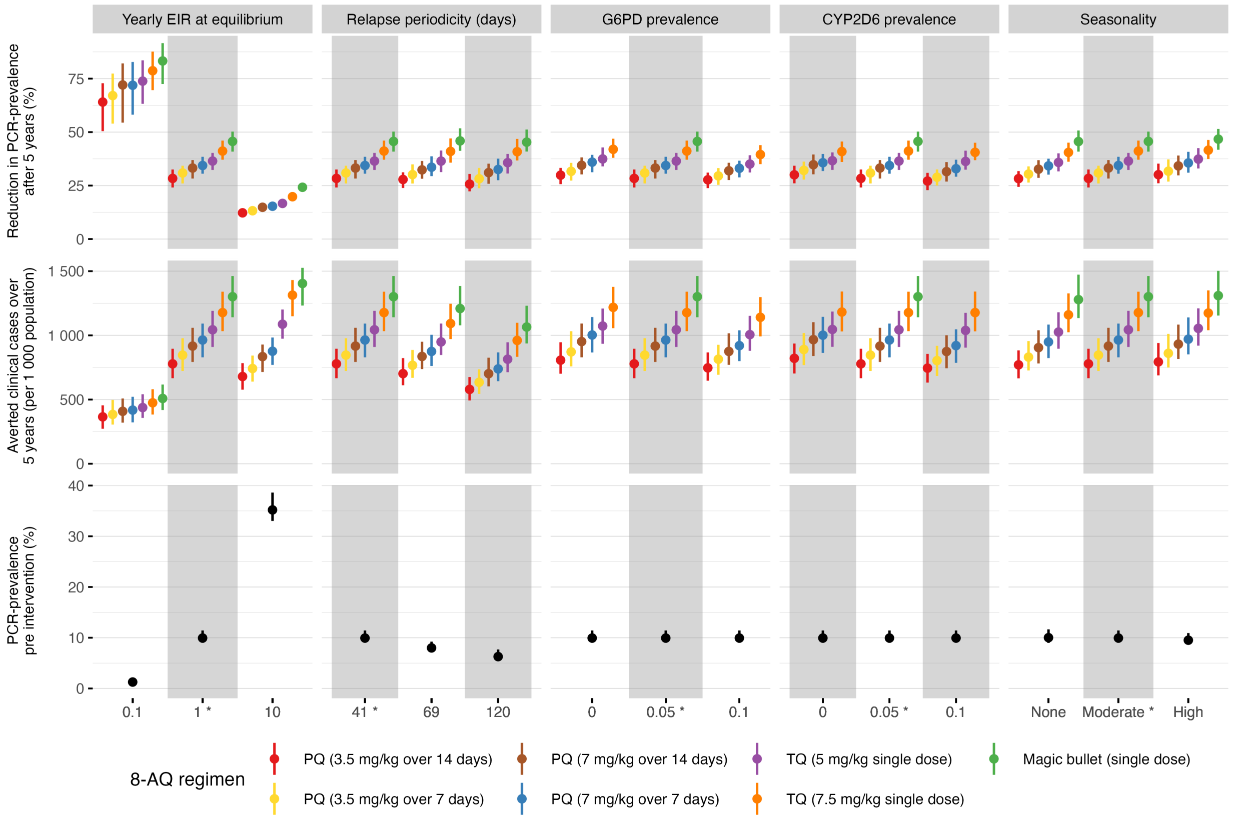

Figure S18: Estimates of radical cure impact obtained by varying key characteristics of the transmission setting. Default values (denoted with an asterisk *) and alternative values are listed in Table S10. Medians and 95% confidence intervals of 100 independent simulations per scenario using the P. vivax Individual-Based Model (PvIBM). All patients were assumed to be treated with chloroquine (CQ) for blood-stage activity. (8-AQ) 8-aminoquinoline, (PQ) primaquine, (TQ) tafenoquine.

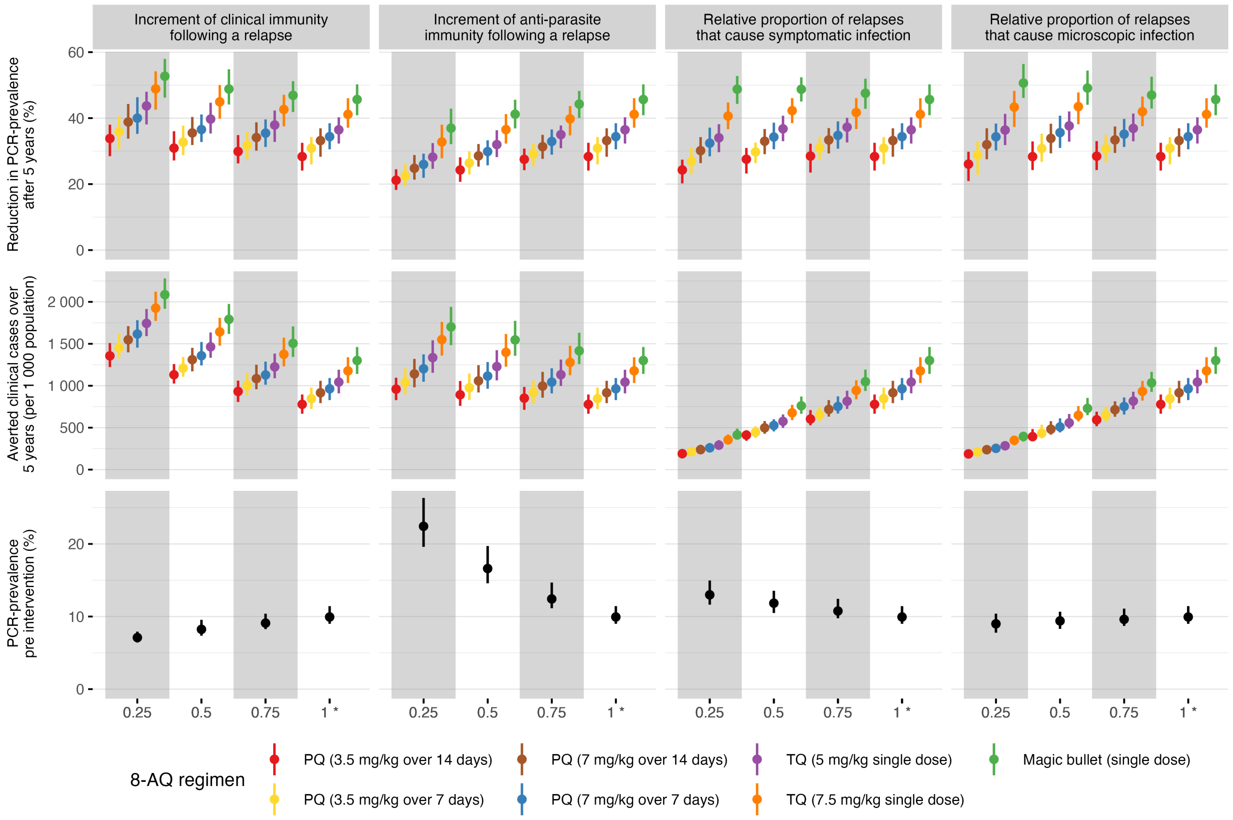

Figure S19: Estimates of radical cure impact obtained by varying key parameters describing the immune system response to relapse infections. Default values (denoted with an asterisk *) and alternative values are listed in Table S10. Medians and 95% confidence intervals of 100 independent simulations per scenario using the P. vivax Individual-Based Model (PvIBM). All patients were assumed to be treated with chloroquine (CQ) for blood-stage activity. (8-AQ) 8-aminoquinoline, (PQ) primaquine, (TQ) tafenoquine.

References

1 Taylor WRJ, Thriemer K, von Seidlein L, *et al.* Short-course primaquine for the radical cure of Plasmodium vivax malaria: A multicentre, randomised, placebo-controlled non-inferiority trial. *The Lancet* 2019; **394**: 929–38.

2 Commons RJ, Rajasekhar M, Edler P, *et al.* Effect of primaquine dose on the risk of recurrence in patients with uncomplicated Plasmodium vivax: A systematic review and individual patient data meta-analysis. *The Lancet Infectious Diseases* 2023; **24**: 172–83.

3 Watson JA, Commons RJ, Tarning J, *et al.* The clinical pharmacology of tafenoquine in the radical cure of Plasmodium vivax malaria: An individual patient data meta-analysis. *eLife* 2022; **11**: e83433.

4 Huber JH, Koepfli C, España G, Nekkab N, White MT, Alex Perkins T. How radical is radical cure? Site-specific biases in clinical trials underestimate the effect of radical cure on Plasmodium vivax hypnozoites. *Malaria Journal* 2021; **20**: 479.

5 Bennett JW, Pybus BS, Yadava A, *et al.* Primaquine Failure and Cytochrome P-450 2D6 in *Plasmodium Vivax* Malaria. *New England Journal of Medicine* 2013; **369**: 1381–2.

6 St Jean PL, Xue Z, Carter N, *et al.* Tafenoquine treatment of Plasmodium vivax malaria: Suggestive evidence that CYP2D6 reduced metabolism is not associated with relapse in the Phase 2b DETECTIVE trial. *Malaria Journal* 2016; **15**: 97.

7 Silvino ACR, Costa GL, Araújo FCF de, *et al.* Variation in Human Cytochrome P-450 Drug-Metabolism Genes: A Gateway to the Understanding of Plasmodium vivax Relapses. *PLOS ONE* 2016; **11**: e0160172.

8 Koopmans AB, Braakman MH, Vinkers DJ, Hoek HW, Van Harten PN. Meta-analysis of probability estimates of worldwide variation of CYP2D6 and CYP2C19. *Translational Psychiatry* 2021; **11**: 141.

9 White MT, Karl S, Battle KE, Hay SI, Mueller I, Ghani AC. Modelling the contribution of the hypnozoite reservoir to Plasmodium vivax transmission. *eLife* 2014; **3**: 1–19.

10 Karunajeewa HA, Mueller I, Senn M, *et al.* A Trial of Combination Antimalarial Therapies in Children from Papua New Guinea. *New England Journal of Medicine* 2008; **359**: 2545–57.

11 Lacerda MVG, Llanos-Cuentas A, Krudsood S, *et al.* Single-Dose Tafenoquine to Prevent Relapse of Plasmodium vivax Malaria. *New England Journal of Medicine* 2019; **380**: 215–28.

12 Vats D, Flegal JM, Jones GL. Multivariate output analysis for Markov chain Monte Carlo. *Biometrika* 2019; **106**: 321–37.

13 Nekkab N, Lana R, Lacerda M, *et al.* Estimated impact of tafenoquine for Plasmodium vivax control and elimination in Brazil: A modelling study. *PLOS Medicine* 2021; **18**: e1003535.

14 White MT, Walker P, Karl S, *et al.* Mathematical modelling of the impact of expanding levels of malaria control interventions on Plasmodium vivax. *Nature Communications* 2018; **9**: 3300.

15 White MT, Griffin JT, Churcher TS, Ferguson NM, Basáñez M-G, Ghani AC. Modelling the impact of vector control interventions on Anopheles gambiae population dynamics. *Parasites & Vectors* 2011; **4**: 153.

16 Carnevale P, Frezil JL, Bosseno MF, Le Pont F, Lancien J. The aggressiveness of Anopheles gambiae A in relation to the age and sex of the human subjects. *Bulletin of the World Health Organization* 1978; **56**: 147–54.

17 Port GR, Boreham PFL, Bryan JH. The relationship of host size to feeding by mosquitoes of the Anopheles gambiae Giles complex (Diptera: Culicidae). *Bulletin of Entomological Research* 1980; **70**: 133–44.

18 Kiattibutr K, Roobsoong W, Sriwichai P, *et al.* Infectivity of symptomatic and asymptomatic Plasmodium vivax infections to a Southeast Asian vector, Anopheles dirus. *International Journal for Parasitology* 2017; **47**: 163–70.

19 Herrera S, Solarte Y, Jordán-Villegas A, *et al.* Consistent safety and infectivity in sporozoite challenge model of Plasmodium vivax in malaria-naive human volunteers. *The American journal of tropical medicine and hygiene* 2011; **84**: 4–11.

20 Pukrittayakamee S, Imwong M, Singhasivanon P, Stepniewska K, Day NJ, White NJ. Effects of different antimalarial drugs on gametocyte carriage in P. Vivax malaria. *The American journal of tropical medicine and hygiene* 2008; **79**: 378–84.

21 Pal S, Bansil P, Bancone G, *et al.* Evaluation of a Novel Quantitative Test for Glucose-6-Phosphate Dehydrogenase Deficiency: Bringing Quantitative Testing for Glucose-6-Phosphate Dehydrogenase Deficiency Closer to the Patient. *The American Journal of Tropical Medicine and Hygiene* 2019; **100**: 213–21.

22 Almeida ED, Rodrigues LCS, Vieira JLF. Estimates of adherence to treatment of vivax malaria. *Malaria Journal* 2014; **13**: 321.

23 WHO. WHO Guidelines for malaria. WHO, 2023 https://www.who.int/publications/i/item/guidelines-for-malaria (accessed July 17, 2023).

24 Global Malaria Programme. Guide to G6PD deficiency rapid diagnostic testing to support P. Vivax radical cure. Geneva: World Health Organization, 2018 https://www.who.int/publications/i/item/9789241514286 (accessed June 21, 2022).

25 Rueangweerayut R, Bancone G, Harrell EJ, *et al.* Hemolytic Potential of Tafenoquine in Female Volunteers Heterozygous for Glucose-6-Phosphate Dehydrogenase (G6PD) Deficiency (G6PD Mahidol Variant) versus G6PD-Normal Volunteers. *The American Journal of Tropical Medicine and Hygiene* 2017; **97**: 702–11.

26 Killeen GF, McKenzie FE, Foy BD, Schieffelin C, Billingsley PF, Beier JC. A simplified model for predicting malaria entomologic inoculation rates based on entomologic and parasitologic parameters relevant to control. *The American Journal of Tropical Medicine and Hygiene* 2000; **62**: 535–44.
